# Employing Constant Rate Filtration To Assess Active Pharmaceutical Ingredient Washing Efficiency

**DOI:** 10.1021/acs.oprd.1c00272

**Published:** 2021-12-21

**Authors:** Muhid Shahid, Chloé Faure, Sara Ottoboni, Leo Lue, Chris Price

**Affiliations:** † EPSRC Continuous Manufacturing & Advanced Crystallisation (CMAC) Future Manufacturing Research Hub, University of Strathclyde, Glasgow G1 1XQ, U.K.; ‡ Département de Genie Chimique-Génie des Procédés, UT Paul Sabatier, 137 Avenue de Rangueil, BP 67701, 31077 Toulouse Cedex 4, France; § Department of Chemical and Process Engineering, University of Strathclyde, Glasgow G1 1XQ, U.K.

**Keywords:** constant rate filtration, washing, impurity precipitation, antisolvent crystallization, agglomeration

## Abstract

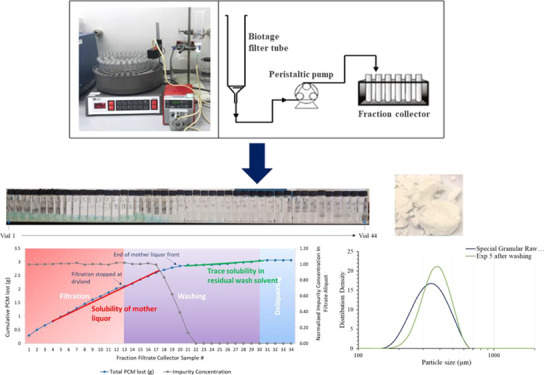

Washing is a key step in pharmaceutical isolation to remove unwanted crystallization solvents and dissolved impurities (mother liquor) from the active pharmaceutical ingredient (API) filter cake to ensure the purity of the product whilst maximizing yield. It is therefore essential to avoid both product dissolution and impurity precipitation during washing, especially precipitation of impurities caused by the wash solvent acting as an antisolvent, affecting purity and causing agglomerate formation. This work investigates the wash solvent flow through a saturated filter cake to optimize washing by displacement, taking account of diffusional mechanisms and manipulating the wash contact time. Constant rate filtration/washing is employed in this study using readily available laboratory equipment. One advantage of using constant rate filtration in this work is that it allows for the collection of separate aliquots during all stages of filtration, washing, and deliquoring of the API cake. This enables a wash profile to be obtained, as well as providing an overall picture on the mass of API lost during isolation and so can assist in optimizing the washing strategy. Particle size analysis of damp cake obtained straight after washing is also performed using laser diffraction. This allowed for agglomerate formation caused during washing to be distinguished from agglomeration that would be caused by subsequent drying of the wet filter cake. This work aims at improving pharmaceutical product quality, increasing sustainability, and reducing manufacturing cost.

## 1 Introduction

In the pharmaceutical industry, the final drug substance (active pharmaceutical ingredient, API) and the key synthetic intermediates are mostly isolated as crystalline solids.^1^ A considerable amount of effort is spent in the crystallization process to produce a crystalline solid with the requisite chemical quality together with the right physical properties (filterability, product size, uniformity, and so forth) for isolation and further downstream processing to manufacture the drug product.^2^ Whilst carefully designed upstream processes may attain the desired crystal properties in suspension, these are often compromised during the isolation of the API by filtration, washing, and drying. These isolation processes pose significant challenges to the production of crystals with the desired physical properties, avoiding granulating, breaking the +crystals, or precipitating dissolved products and impurities.^3^

The pharmaceutical industry sets a high standard of purity, which the final API must meet.^4^ To achieve this, washing of the cake is a fundamental postfiltration treatment step. After filtration, residual mother liquor (crystallization solvent containing unreacted starting materials and unacceptable side products) is retained and trapped inside the porous structure of the solid bed. If this mother liquor is not removed before the downstream drying process, the dissolved material will be deposited on the product crystal surfaces, resulting in the presence of impurities in the final product.^5^ Therefore, washing is a vital purification step, which is required to remove impurities from the filtered cake.

Washing of the cake is typically achieved using the same driving force (centrifugal, pressure, and vacuum) as filtration and is carried out in the same process equipment used for filtration, so usually little or no additional equipment is required.^6^

Filtration can either be carried out using a constant pressure driving force or a constant rate filtration. Most laboratory filtration works to isolate the product using constant pressure, normally using laboratory vacuum or overpressure from a compressed gas line.^7^ This is largely due to the readily available laboratory filtration equipment designed with this in mind.^8^

There are a number of advantages of using constant rate filtration in preference to constant pressure filtration. The homogeneous constant growth rate of the cake, using constant rate filtration, provides a better packing structure independent of cake thickness.^9−11^ Constant rate filtration allows the rate of liquid transport through the filter medium to be fixed, whereas in constant pressure filtration, the initial rate of flow through the filter medium is the highest when the cake is thin, but declines as the cake builds and the filter cake resistance increases. As a consequence, any fine particles present are less likely to be carried into the filter medium, leading to filter medium blinding. Reports by many researchers indicate that the measured cake and medium resistances are influenced by the migration of fine particles accumulating in the lower layers of the cake close to and within the filter, reducing the flow rate through them.^12−18^

This work investigates the use of constant rate filtration for improving washing of saturated API cake. The aim of this work is to use constant rate filtration to design a washing strategy that is effective and reproducible in obtaining washed material with the required purity, yield, and particle size distribution (PSD), whilst minimizing waste generation.

## 2 Theoretical Background

**Figure 1:**
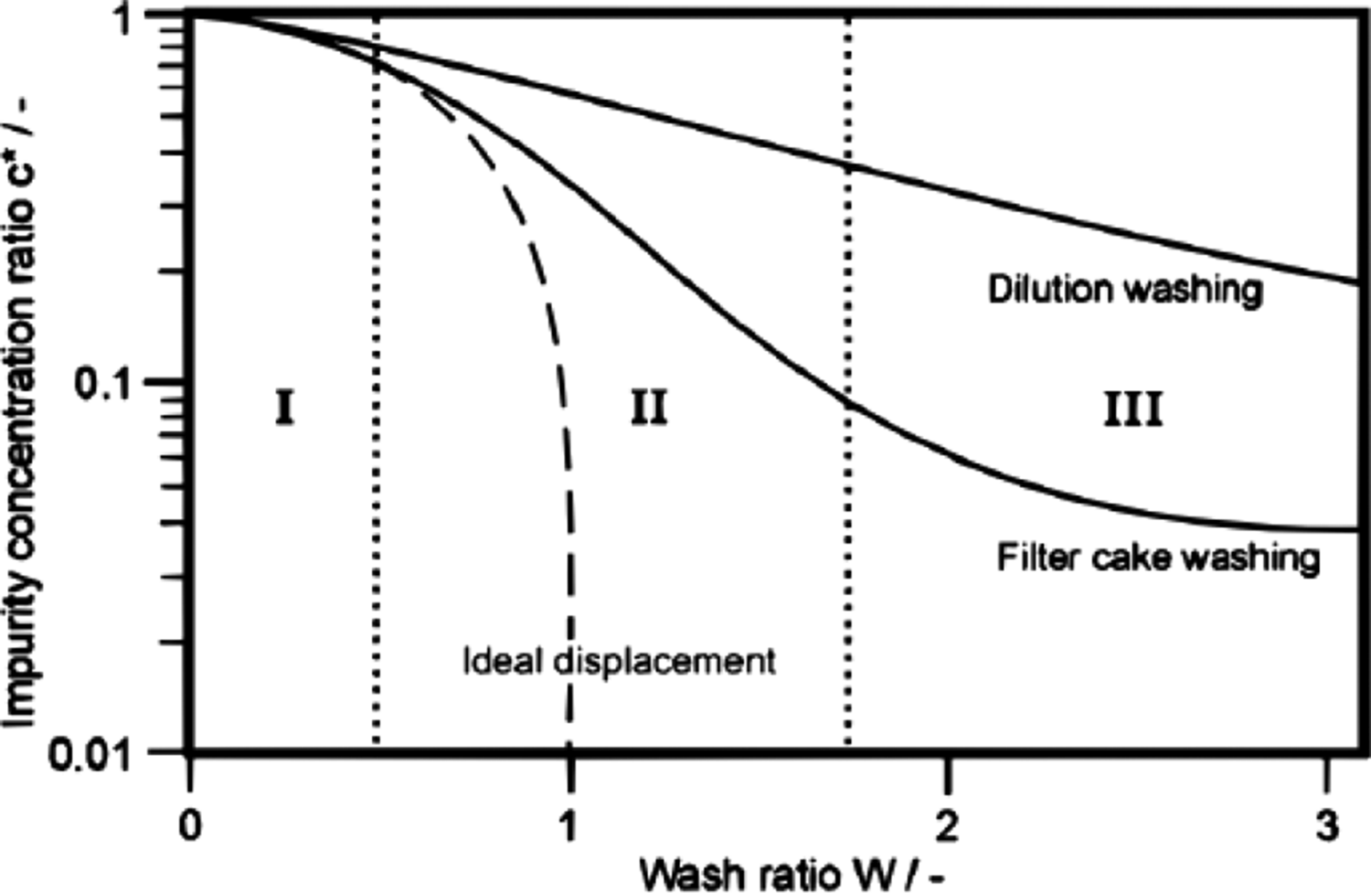
Wash curve obtained from measuring the solute concentration of the filtrate.^20^ [Reproduced with permission from reference 5160391414737, Copyright 2007, Elsevier].

After the filtration process, the impurity-laden mother liquor with the dissolved product at or slightly in excess of the equilibrium solubility is removed from the voids in the saturated cake during washing by displacing them using a clean wash solvent. One of the ways of measuring the cake washing process is by determining the solute (the impurity and the API) concentration of the collected filtrate as a function of the wash ratio, where the wash ratio is defined as the volume of the wash solvent used divided by the volume of the mother liquor trapped in the cake at the start of the washing process. For the solute concentration, the remaining impurity concentration ratio, c*, is used (eq 1):
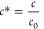
1where the remaining impurity concentration (*c*) is related to the initial concentration (*c*_0_). Figure 1 shows three idealized washing curves obtained, which can be divided into three main regions. (I) is the initial displacement region (a plug-flow regime), where the residual mother liquor is removed from the larger pores because of the wash solvent entrance. (II) is the intermediate stage, where the direct displacement of the mother liquor occurs in the smaller pores and the wash solvent starts to dilute the mother liquor as it passes through the larger pores, and hence, the mass-transfer process starts. (III) is the mass-transfer region, where diffusion is the rate-limiting step as the mother liquor diffuses from the fine pore structure into the wash solvent over the entire volume of the cake.^19^

In an ideal case, the wash solvent would run in a plug-flow regime, displacing all the mother liquor from the cake, as is the case in region I. This is due to the high efficiency of the ideal displacement regime, and hence, a small amount of wash solvent is required. However, ideal displacement can never be reached in real systems where there is a distribution of the particle size rather than a perfect monosized product; in practice, a combination of displacement, dilution, and diffusion washing mechanism is required for effective washing.

The relative importance of each regime in a given experiment depends on the physical operating conditions, solvent properties, and the microstructure of the pores in the filter cake influencing the local wash liquid flow rate.^4,10,19^ The particle morphology (shape) and size distribution therefore affect washing performance. Cakes formed of larger particles contain larger pores and wider pathways, which reduces the specific resistance of the cake and enables higher wash flowrates.^12^ Cakes formed with fine particles and broad PSD have smaller pore networks, lower permeability, and hence, filtration times are extended. Mass-transfer and diffusion mechanisms play a larger role in the washing regime of such filter cakes. Further negative effects such as local under saturation of the wash medium or the formation of cracks or craters due to malformation of the cake can lead to liquid bypassing or channeling (especially when a high driving force is applied).^20^

Generally, washing is performed immediately after filtration to avoid the cake surface from beginning to dry. The ideal starting condition is a well-formed filter cake with a level surface, which is fully saturated with mother liquor.^20,21^ The wash solvent is carefully distributed over the top of the cake, preferably using a misting spray, as a disturbance to the cake surface can lead to thin spots and cracks, resulting in the wash bypassing areas as it follows the path of the least resistance.

The selection of the wash solvent is an essential part of the API cake washing process. It is important to pick a solvent that will minimize dissolution of the API crystals, whilst also avoiding any precipitation of both dissolved API and impurities when the wash solvent comes into contact with the retained crystallization solvent in the saturated filtered cake. Selecting a wash solvent to avoid both these phenomena can be challenging, but is essential to maintain yield, purity, and particle characteristics of the crystals obtained through the crystallization processes.

A screening methodology was developed in a previous study to qualitatively and quantitatively analyze the propensity for precipitation to occur during the washing of an API (paracetamol, PCM) with different solvents.^22,23^ This methodology allowed us to identify solvent combinations that would prevent/limit any precipitation or dissolution in the case of PCM. It was found that starting the washing process with the mixture consisting of both a well-chosen wash solvent and the crystallization solvent is best for avoiding precipitation during the initial stages of washing. This is followed by washing with the selected wash solvent in which the API has negligible solubility, leading to a process where there is a reduced chance of agglomerate formation during drying. Although the methodology is exemplified with PCM, the approach is likely to be widely applicable.

This work used the selected wash solvents from the previous work^22^ and used constant rate filtration to investigate wash performance for PCM as a representative API. Three different grades of PCM were used to perform the investigation of the effect of particle size on washing performance. Constant rate filtration, and subsequently constant rate washing, was employed in this study to investigate whether the particle packing and cake structure formed using this method allows for more uniform migration of the wash solvent through the API cake and therefore leads to improved washing performance. Furthermore, unlike in constant pressure filtration, the wash solvent flowrate through the saturated cake can be controlled in constant rate filtration. This enables the role of wash solvent contact time to be investigated using very small quantities of the material, consistent with that typically available in early pharmaceutical development.

## 3 Materials and Methods

### 3.1 Raw Materials

PCM was selected as a representative test compound with three different size distributions (micronized, crystalline, and granular) being used. The micronized-grade material (batch 042213E407; Mallinckrodt Inc., Staines-upon-Thames, U.K.) settles very slowly from suspension and has a large, wetted surface area to wash. The granular material (batch 161,713 J561; Mallinckrodt Inc.), on the other hand, settles rapidly and has a wide PSD. The intermediate grade (batch 637514D001; Mallinckrodt Inc.) is more typical of the size distribution of a pharmaceutical crystalline material. PSD (PSD) determined for all three PCM grades investigated is given in Table 1.

**Table 1:** PSD of Different PCM Grades Investigated within This Study

PCM grades	PSD		
	*D*_10_ (μm)	*D*_50_ (μm)	*D*_90_ (μm)
micronized	6.52	27.6	198.2
crystalline	12.48	43.9	101.3
granular	246.8	361.4	517.8

Patent Blue V sodium salt (LOT: BCBP1872V; Sigma-Aldrich) was used as an impurity in the study to help evaluate wash performance and cake purity. The dye aids visualization of the filtration and washing process as well as being readily quantified spectroscopically.

To investigate the washing efficiency of a “real process” slurry, three different crystallization solvents, commonly used in industry, were used: ethanol (absolute, purity ≥99.8%, Sigma-Aldrich), isopropanol (purity ≥99.5%, Sigma-Aldrich), and 3-methylbutan-1-ol (also known as isoamyl alcohol) (purity ≥99%, Sigma-Aldrich). Three wash solvents used in this study were acetonitrile (purity ≥99.9%, Sigma-Aldrich), *n*-heptane (purity 99.9%, Sigma-Aldrich), and *n*-dodecane (purity 99%, Alfa Aesar). Acetonitrile was chosen because of the relatively high solubility of the API compared to that in both n-heptane and *n*-dodecane. *n*-Dodecane is immiscible in all three crystallization solvents; hence, it is useful for investigating washing displacement mechanisms. Also, in some experiments using *n*-heptane or *n*-dodecane, a wash solution consisting of a mixture of the crystallization and the wash solvent was used as a first wash before using the pure wash solvent as a second wash (the composition ratios of the solvent mixtures were taken from the results of previous work).^22^ This enabled the examination of the approach for the minimization of “antisolvent” effects taking place during washing. For acetonitrile, no “antisolvent” effect was identified during previous studies, and hence, only pure acetonitrile was used as a first wash in all experiments containing acetonitrile.

For postexperimental analysis, 2,2,4-trimethylpentane (isooctane) (purity 99.9% (GC), Merck) is used as a wet dispersant for particle size analysis of the input PCM grades as well as the final washed cake. Deuterated dimethyl sulfoxide (DMSO-d_6_) (extent of deuteration, 99.8%, for NMR spectroscopy, VWR) was used for NMR analysis of the washed cake to quantify the amount of mother liquor present in the final API product.

### 3.2 Suspension Preparation

PCM particle suspension was prepared by adding the API in two stages: the first portion was to form a saturated solution at the laboratory temperature (22 °C). A known mass of Patent Blue V dye was then added to the saturated solution (below the solubility limit of each crystallization solvent), before a second portion of PCM was added to the saturated solution to form a crystal suspension with 15% by mass, solid loading. In this way, minimal change to the PSD of the raw material was ensured.

The solubility of PCM and Patent Blue V dye in all the solvents used throughout this work was taken from the literature where available,^24^ and it is also determined experimentally by gravimetric analysis. A Hailea HC-100A chiller was used to maintain the temperature at 22 °C (the average temperature of the laboratory, where the antisolvent screening experiments were conducted). Excess PCM/Patent Blue V dye was added to 20 mL, clear glass vials together with the solvent and a magnetic stirrer bar. The vials were sealed and left on a multiposition stirrer plate inside the water bath for around 48 h to equilibrate. Samples of the solutions were then taken from the slurry in the vials using a syringe and filtered using a PES syringe filter (Fisher brand, Cat No. 15206869, 0.2 μm, sterile), and added to a separate glass vial which was weighed and then left to dry. Table 2 shows the determined solubility in each solvent investigated, which is then used to prepare the PCM slurry with blue dye impurity.

**Table 2:** Solubility Measurements Determined Experimentally at 22 °C (Laboratory Temperature)

solvent	PCM solubility (mg/g solvent)	blue dye solubility (mg/g solvent)
ethanol	186.8	1.027
isopropanol	114.1	0.701
isoamyl alcohol	52.6	0.736
acetonitrile	24.03	0.418
*n*-heptane	0.267	0.000
*n*-dodecane	0.072	0.000

### 3.3 Experimental Setup and Design

**Figure 2:**
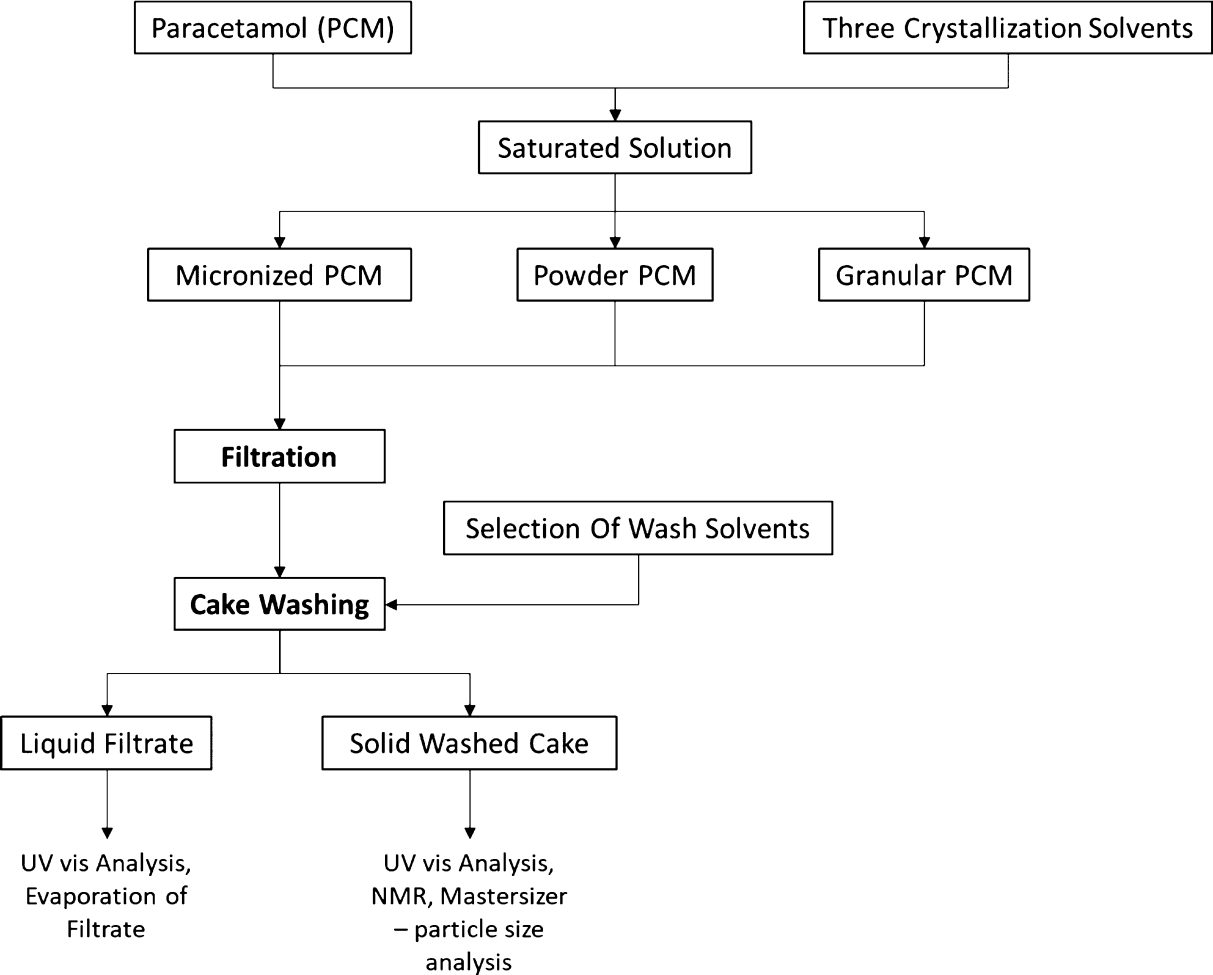
Experimental procedure.

In order to investigate washing performance and the propensity of different grades of PCM to agglomerate under different process conditions, the experimental procedure was divided into a series of consecutive steps, as can be seen in Figure 2.

A multivariate design of experiment (DoE) approach was used to investigate the combined effects of the key parameters on the final quality of the washed API cake. MODDE (Umetrics, Sweden) was the software used for the DOE analysis. For this work, a D-optimal screening approach was used in order to minimize the number of experiments whilst maximizing the insight gained, this resulting in 22 experiments with 3 center point experiments to determine the reproducibility of the experimental procedure (see the Supporting Information).

The D-optimal approach used in this work is appropriate because the experimental variables investigated comprised a combination of quantitative and qualitative factors.^25^Table 3 contains the list of variables and responses used within this study. A number of potential factors were kept constant and were not included in the DOE: the API used (PCM), the impurity used (Patent Blue V dye), the volume of slurry (50 mL), the slurry solid loading (15% w/w), the pore size of filter media used (nominal pore size of 20 μm), the temperature of the suspension and wash solvent during filtration and washing (laboratory temperature ≈ 20 °C).

**Table 3:** Table of Factors, Responses, and Analytical Techniques Used To Quantify the Responses in the DoE

variables	
factors (abbreviations)	range and units
solid API grade (Par)	micronized, crystalline, granular
crystallization solvent (Cry)	ethanol, isopropanol, isoamyl alcohol
wash solvent (Was)	*n*-dodecane, *n*-heptane, acetonitrile, mix dodecane, mix *n*-heptane
filtration and washing rate (Fil)	10–100 rpm (1.3–11.7 mL/min, respectively)
volume of the wash solvent (Vol)	1 void volume^a^, 2 void volume, 3 void volume
number of washes (Num)	1, 2, 3

responses	
responses (abbreviation)	analytical method used to quantify response
mother liquor remaining (MLR)	^1^H NMR for residual solvent composition
impurity removal (IR)	UV–vis spectrophotometer
API lost to washing (APIL)	mass balance
change in *D*_10_ (*D*_10_)	particle size analyzer, Mastersizer 3000
change in *D*_50_ (*D*_50_)	particle size analyzer, Mastersizer 3000
change in *D*_90_ (*D*_90_)	particle size analyzer, Mastersizer 3000

a Void volume refers to the wash volume quantity that corresponds to cake pore volume. It can be calculated using eq 2.

Coefficient plots are used in the Results and Discussion section to report the correlation between factors and responses. Coefficient plots provide the graphical representation of the significance of the model terms in explaining each experimentally determined response. A significant term is one with a large distance from *y* = 0 as well as having an uncertainty level that does not extend across the *y* = 0 value. The error bar represents the 95% confidence interval related to the coefficient. Some of the regression coefficient plots presented in the Results and Discussion section reports on the *y*-axis (responses) employ the expression “extended.” If a term in the model comprises a qualitative factor, C, with *x* levels, there will be *x* – 1 expanded terms associated with that term for the regular option, whereas in the expanded option, all of the levels are correlated with the selected response. For example, considering the API grade as a qualitative factor, there are three levels: micronized, crystalline, and special granular. In the regular option for presenting the qualitative coefficients, MODDE plots report crystalline and special granular, while with the expanded option MODDE plots all of the three levels.^25,26^

The combination of qualitative and quantitative factors used in this DoE design does not allow for the prediction of optimal design space. However, the use of qualitative factors was necessary to screen for essential washing parameters. Therefore, the results from the coefficient plot were used together with the experimental observations to qualitatively define optimal washing design space for PCM API with the blue dye impurity.

**Figure 3:**
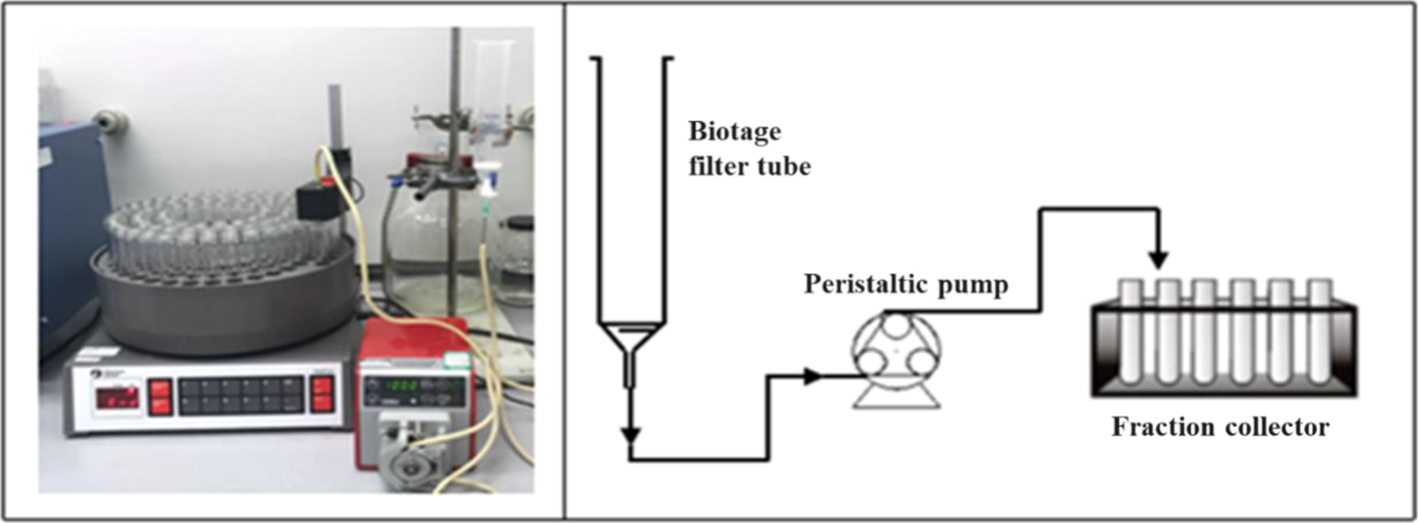
Experimental setup for constant rate filtration, including the process flow diagram of the setup used.

Figure 3 shows the experimental setup used in the laboratory as well as a process flow diagram of the setup. For filtration and washing, a Biotage ISOLUTE (Biotage AB, Uppsala, Sweden) 70 mL single-fritted polypropylene reservoir with 20 μm pore size was used, and this was connected to a polytetrafluoroethylene (PTFE) valve and a flexible tube (Watson-Marlow, Marprene tubing, 302.0016.016#14, 1.6 mm Bore × 1.6 mm wall, volume hold up 2 mL/m). The tube was connected to a peristaltic pump (Watson-Marlow, 120 pump drive, 40DM3 pump head) that controlled the flow of solvent. A fraction collector (RediFrac, Code No. 18-1003-64, GE Healthcare Bio-Sciences AB, Sweden) was placed at the end of the liquid discharge tube to segregate and collect the different fractions of the filtrate, which were later analyzed by UV–vis spectrophotometry to obtain a high-resolution time-resolved washing profile. The volume of the individual fractions collected for each experiment was kept consistent at around 3.2 mL by adjusting the filtrate collection time, depending on the pumping rate (Table 3).

Before the start of each experiment, the filter tube and filtrate collection vials were weighed. A complete mass balance was maintained throughout the experiment. A 50 mL sample of crystal suspension was transferred to the filter tube, making sure complete removal of slurry was achieved while transferring from the sample bottle to the biotage filter tube. The peristaltic pump was then turned on at the required pumping rate immediately before opening the valve and allowing the filtrate to flow through the filter medium into the collection vials. Upon reaching the dryland (the point when the filter cake surface is first exposed), the pump was halted, the PTFE valve closed, the filter tube with the valve was weighed, and the filter cake thickness was measured. The required volume of the wash solvent was then measured and carefully added to the filter tube by slowly running the wash solvent down the wall of the tube, making sure not to disturb the filter cake surface. The wash volume corresponds to the cake pore volume (eq 2):^27,28^
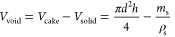
2where *d* is the cake diameter (m), *h* is the cake height (m), *m*_s_ is the mass of the API in the filter cake (kg), and ρ_s_ is the crystallographic particle density of the API (kg/m^3^). The role of wash volume is investigated in this study by adjusting both the wash quantity relative to cake void volume and the number of washes used, see Table 3.

The wash solvent passes through the cake and the medium at the same pumping rate as the filtration stage of the experiment. This procedure is repeated for each washing step if more than one was required. The final wash is followed by cake deliquoring which is stopped as soon as the bubble point is detected (the point at which a break in the steady flow of filtrate is observed). The filter tube mass and the filter cake thickness are then measured and the vial numbers noted at the points where filtration and washing stops.

### 3.4 Liquid Filtrate Offline Post Analysis

**Figure 4:**
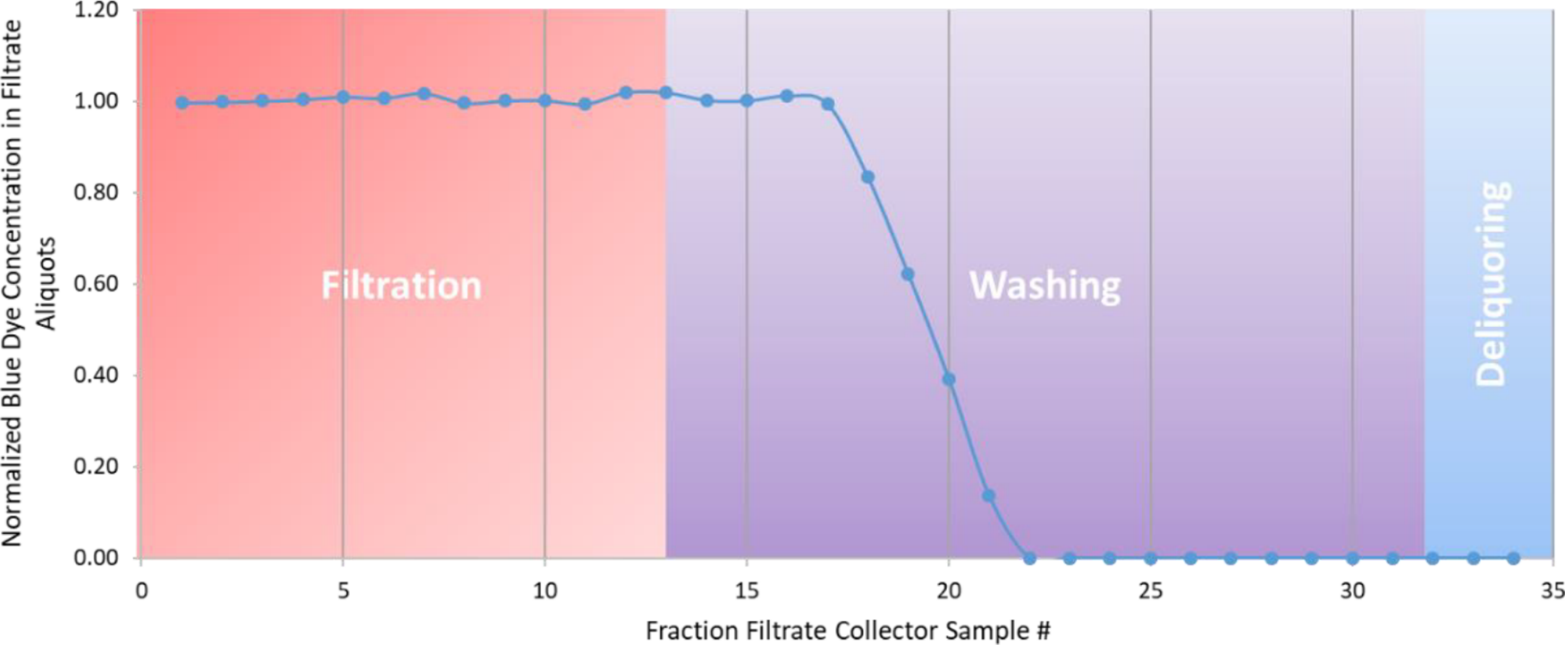
Evolution of blue dye impurity concentration in filtrate – EXP 1.

At the end of the experiment, the vials containing the filtrate are weighed. The filtrate sample is then analyzed with UV–vis spectroscopy to quantify the Patent Blue V dye. This allows for the high-resolution time-resolved wash profile of the experiment to be obtained. The impurity removal performance is calculated by determining the number of collected vials of filtrate taken to remove the blue dye and for the blue dye concentration to level off at its minimum value. An example wash profile obtained from experiment 1 is presented in Figure 4. From the wash profile, it is apparent that the blue dye concentration remains constant for a period of time after the filtration reaches dryland and the wash solvent is transferred to the filter cake. The concentration starts to decrease at filtrate sample number 17. This is the point where the first of the wash solvent starts to emerge from the filter cake and is collected in the filtrate vial. The concentration declines and levels off at a final concentration at or close to 0 by filtrate sample number 22. Therefore, the impurity removal response input to the DoE software MODDE for experiment 1 is 6 as it took six filtrate samples for the blue dye concentration to approach zero.

**Figure 5:**
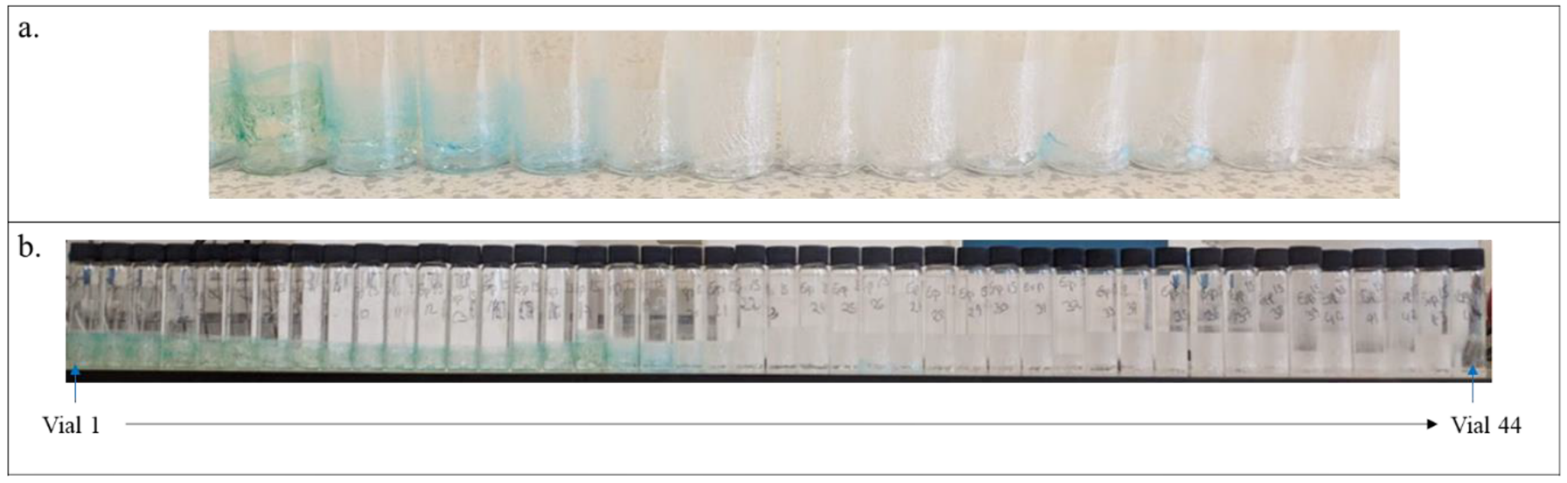
(a) Close-up of a few dried filtrate vials showing the presence of the precipitated material. (b) Collected filtrate vials from experiment 15 showing gradual blue dye impurity removal from the system.

Following the UV–vis analysis, the remaining filtrate in the vials were reweighed, and the vials were left to dry out fully for gravimetric analysis. Determining the amount of API dissolved in each collected filtrate aliquot, together with the knowledge of the quantity of API dissolved in the mother liquor solution at the start of the experiment, allows for the mass of API lost during the washing process to be calculated. This allows the API corresponding profile of API loss to washing to be determined, as shown in Figure 5. The data are also included as a DoE response for each experimental run, Table 3.

### 3.5 Solid API Cake Offline Post Analysis

At the end of the experiment, the mass of the filter tube with the washed API cake is measured before deliquored API cake is removed from the biotage filter tube. A small fraction of the cake is taken and added to around 20 mL of water. Any blue dye impurity still present in the API cake at the end of the washing process dissolves in water. This was analyzed using UV–vis spectroscopy, similar to the method used for liquid filtrate samples.

The PSD of the raw PCM grades, as well as that of the damp-washed cakes obtained at the end of each experiment, was analyzed using wet dispersion laser diffraction (Mastersizer 3000 laser diffraction particle size analyzer with a hydro dispersion unit, Malvern Panalytical, UK). The method parameters used for this study were as follows: measurement duration 10 s, number of measurements 5, obscuration limit 5–20%, stabilization time 30 s, and beam length 2.5 mm. To form the wet dispersions, samples of the raw material and the wet filter cakes were suspended in isooctane because of negligible solubility of PCM in this solvent. The suspensions for analysis were prepared at the end of each experiment. The washed cake was vertically sliced along the axis of the cake to take around a quarter of the cake (making sure not to disturb the whole cake or break any agglomerates) and was carefully dispersed in 50 mL of the isooctane solvent. PSD analysis was then performed to measure any change in the particle size caused by agglomerate formation during the washing process. The wet dispersion particle size analysis approach was selected in preference to dry dispersion to avoid the problem of washed API cake drying out and so forming agglomerates during drying. This way, agglomeration caused by antisolvent effects during the washing process could be analyzed without the effects of drying confounding the analysis.

The change in the particle size response, Table 3, for *D*_10_, *D*_50_, and *D*_90_ is determined using eq 3:
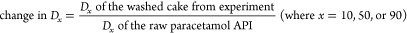
3

To quantify the amount of residual solvent(s) relative to solute, present within the washed cake, a few milligrams of the damp filtered cake was taken and dissolved in 0.75 mL of DMSO-d_6_, for ^1^H-NMR analysis. An AVII+600 NMR Spectrometer BRUKER Advance 2+ (Bruker, UK) is used to collect proton NMR spectra. A T1/T2 relaxation time evaluation is performed for all solvent combinations (process parameters: frequency axis F1 equals to 32, pulse program t1ir, 4 scans, 2 replicas of T1/T2 analysis to evaluate T1 relaxation). Each sample was analyzed in duplicate. This approach allowed the percentage of mother liquor still present in the washed cake (with respect to the quantity of the wash solvent) and hence the mother liquor remaining response to be determined, Table 3.

The remaining damp filter cake is weighed and left to dry in the fume hood to determine the residual solvent content in the cake by loss on drying and the mass of API obtained at the end of the isolation process.

## 4 Results and Discussion

Using the constant rate filtration/washing experimental setup shown in Figure 3 allows for the sequential collection of aliquots of wash filtrate using a fraction collector; this high resolution would be challenging to achieve in a laboratory environment using constant pressure vacuum filtration.^8^ The method used allowed for a detailed analysis of the evolving liquid filtrate composition rather than just the purity of the isolated solid API, which is the part traditionally analyzed during isolation process development.

**Figure 6:**
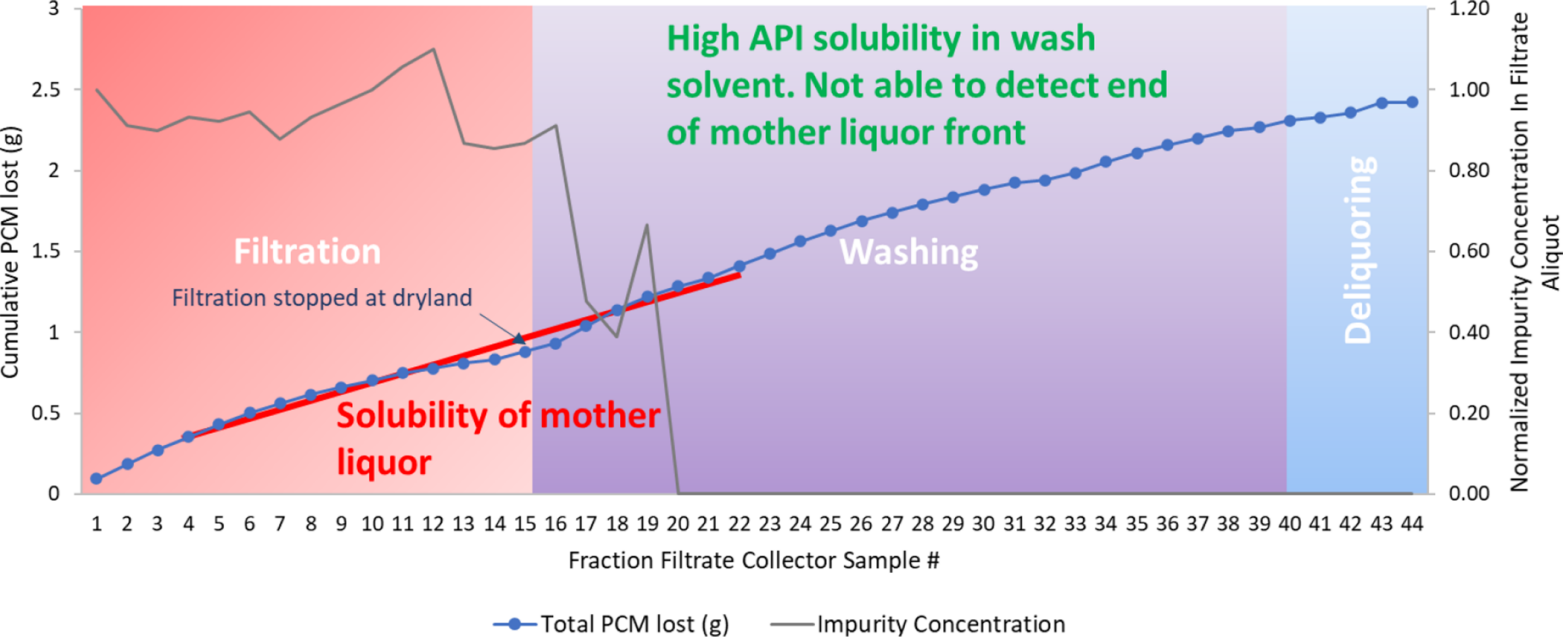
Experiment 15: PCM grade — crystalline, crystallization solvent — isoamyl alcohol, wash solvent — acetonitrile, filtration and washing rate — 100 rpm, volume of wash solvent — 3 cake void volume, number of washes — 3, mass of PCM API lost during wash = 1.48 g.

Figure 5a shows a close-up of a few dried filtrate vials with precipitated PCM present inside with blue dye impurity in some of the vials. Figure 5b shows the sequence of filtrate samples collected during experiment 15. The solution in the vials shows a gradual decrease in color intensity arising from the blue dye impurity removal, with latter vials only showing API removal as all of the blue dye impurity is removed at the start of washing. Figure 6 graphically illustrates the blue dye impurity concentration in the filtrates shown in Figure 5b. In addition, the figure shows the loss of API occurring during washing in experiment 15. The *x*-axis of the graph represents the filtrate sample number in this experiment. The right-hand *y*-axis of the graph corresponds to the normalized blue dye concentration calculated using eq 1 and is represented as gray dots in the graphs. The blue dye concentration seems to decrease after the filtration stage, once all the mother liquor is removed, and reaches zero at filtrate fraction sample 20 as all of the blue dye impurity is removed from the cake. This shows that no further washing of the API cake is required as all of the blue dye is removed, and hence, washing could be stopped at that point without any further usage of the wash solvent. Analyzing the liquid wash filtrate hence allows us to determine the end point of washing, and so could help reduce any wastage of the solvent.

**Figure 7:**
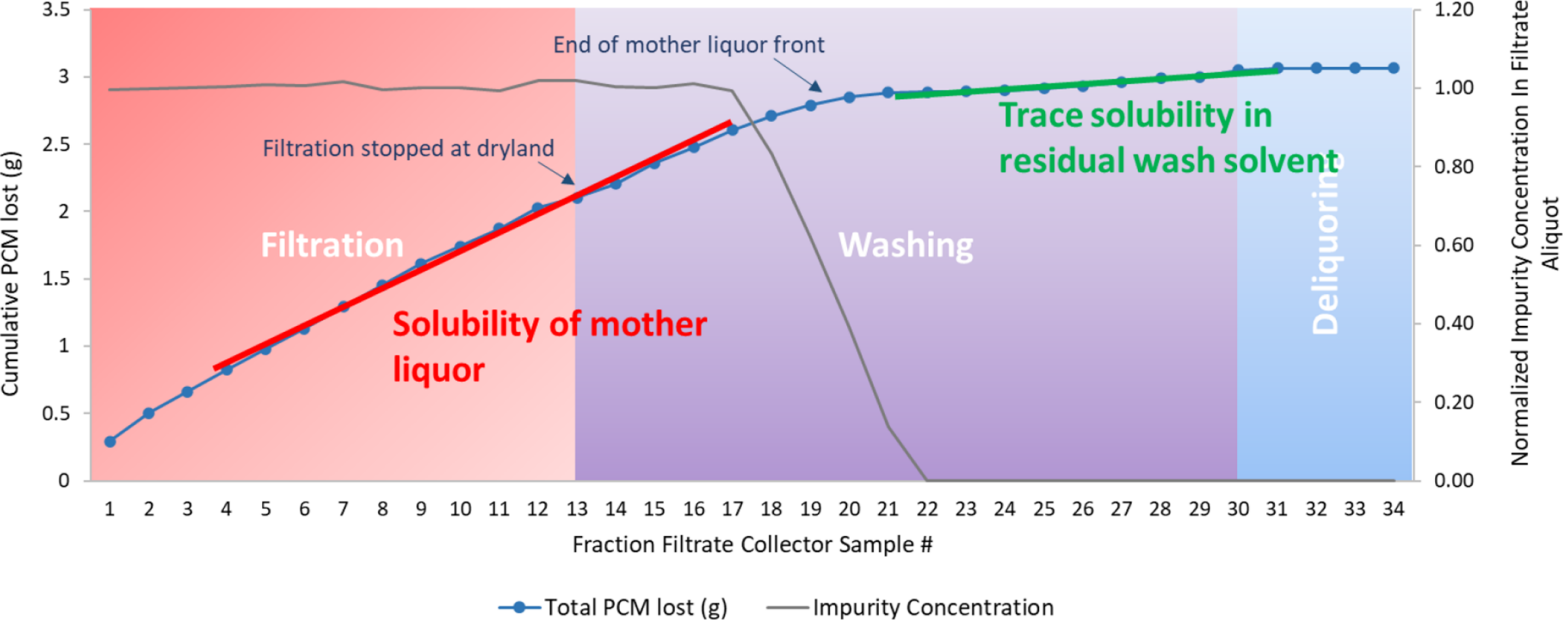
Experiment 1: PCM grade — crystalline, crystallization solvent — ethanol, wash solvent — *n*-heptane, filtration and washing rate — 10 rpm, volume of wash solvent — 1 cake void volume, number of washes — 3, mass of PCM API lost during wash = 0.1 g

The left-hand *y*-axis and the blue dots in Figure 6 correspond to the cumulative API (PCM) lost in solution in the filtrate during the filtration and washing steps in experiment 15. Experiment 15 was carried out using acetonitrile wash solvent, in which PCM has the highest solubility of the three wash solvents used (Table 2). Examining the blue dotted line, there is no difference in the gradient of the line between the filtration and washing step. Comparing this to experiment 1 (Figure 7), we can clearly see a difference in the gradient of the blue dotted line as the mother liquor fronts end and the wash solvent flows through the API-filtered cake and begins to be collected. This is due to n-heptane being used as the wash solvent in experiment 1, in which the API has very low solubility (Table 2). Also, the crystallization solvent in experiment 15 (Figure 6) is isoamyl alcohol, in which PCM has significantly lower solubility than the ethanol crystallization solvent used in experiment 1, Figure 7, (Table 2). Using this constant rate technique for wash process analysis allows us to relate product loss to the combination of solubility in the primary solvent, solubility in the mixture of the primary solvent and wash solvent, and finally solubility in the wash solvent, hence allowing us to minimize the loss of API. Upon seeking to improve the wash efficiency by examining Figures 6 and 7, it is important to note that the API losses associated with displacing mother liquor cannot be reduced by modifying the washing regime, and this is inherently tied to the crystallization process. The opportunity arises from minimizing the API loss associated with dissolution in the mixture of mother liquor and wash solvent and then subsequently in the wash solvent.

### 4.1 API Loss

**Figure 8:**
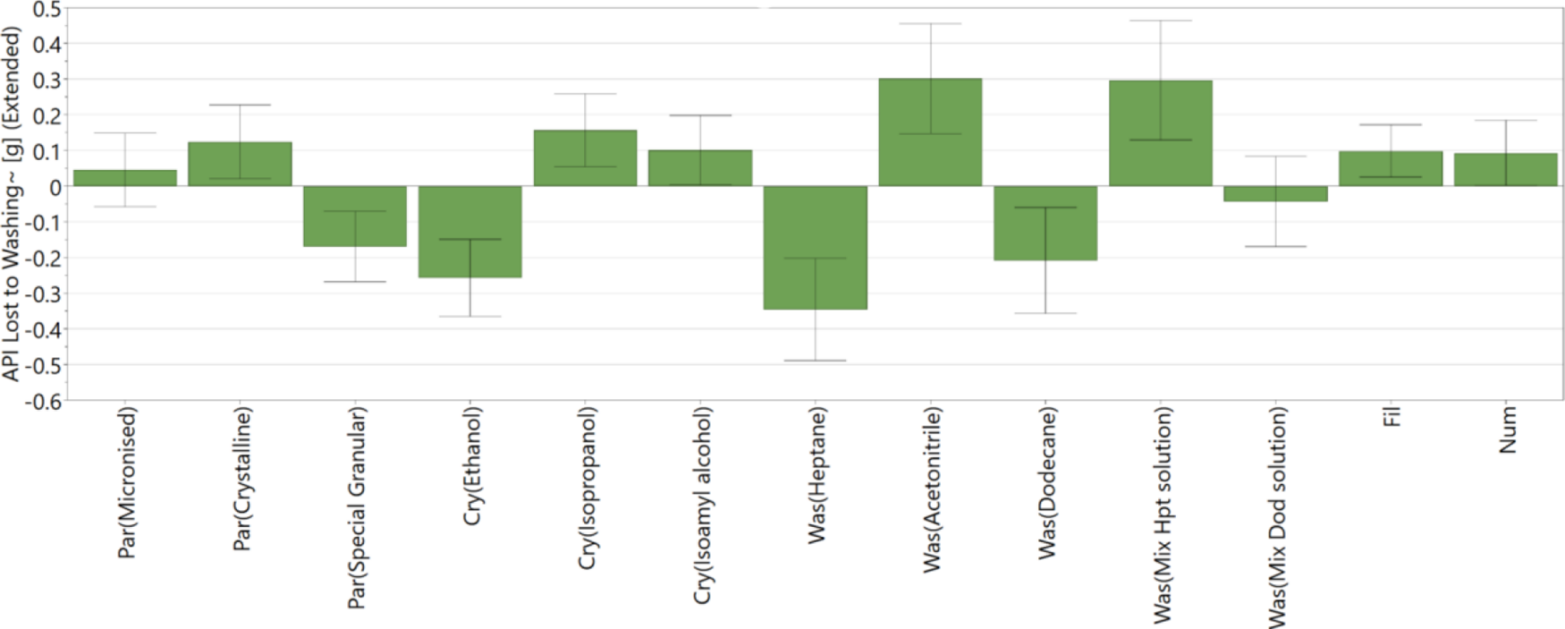
DoE variables that effect API loss during the wash process; *R*^2^ = 0.93, *Q*^2^ = 0.61, and reproducibility = 0.97.

Figure 8 shows how the selected factors affect the API loss during the washing process. The coefficient plot (Figure 8) shows a good reproducibility value of 0.97 and a good fit between the data and the model, and hence, the model has a good capability to predict responses. The plot demonstrates that API loss during washing is affected by factors such as the API grade, the crystallization solvent, the wash solvent, the filtration rate, and the number of washes.

The main factor affecting the API loss during washing is the identity of the wash solvent. Using a wash solvent with high API solubility such as acetonitrile results in a significant amount of API loss during the washing process. The amount of API lost during washing using an n-heptane-crystallization wash solution mixture and an *n*-dodecane-crystallization wash solution mixture is also found to be higher than when using a pure *n*-heptane and *n*-dodecane wash solvent. The binary solvent mixtures have higher solubility than pure wash solvents, and so, the addition of these mixtures as the first wash would result in higher API loss during the washing process.^22^

One of the problems encountered in the initial experimental procedure was the slow rate of filtrate evaporation in experiments containing *n*-dodecane as the wash solvent. The tall 10.5 mL vials with a narrow base (67 mm × 15 mm) that are designed to use with the fraction collector combined with the high boiling point of *n*-dodecane prevented all of the *n*-dodecane from evaporating in some of the filtrate samples even after leaving them in a vacuum oven at high temperatures for over 1 week. This resulted in not being possible to obtain the full mass balance to determine the API lost in the filtrate for experiments containing *n*-dodecane. However, by comparison with experiments where only n-heptane was used as the wash solvent, there was negligible API loss in the filtrate during the final phase of the washing process, where the wash solvent is displaced (such as experiments 8 and 13, Figures S13 and S18 in the Supporting Information). Considering that PCM has similarly negligible solubility in *n*-dodecane, (see Table 2), it was judged to be reasonable to assume similarly that there would be no measurable loss of API in the final filtrate samples collected during washing when using pure *n*-dodecane as the wash solvent.

API grade was another factor affecting API loss during washing. The API grades with small particles, that is, micronized and crystalline PCM have a larger surface area that allows for a greater amount of API to dissolve during washing. The micronized API with a broad particle size results in higher cake tortuosity, resulting in a longer wash solvent flow path through the API cake and so greater chance for API dissolution due to a greater available surface area.^5,12^ Both the filtration/washing rate and the number of washes carried out had an effect on the API loss during washing. The range of wash solvent flowrates investigated in the DoE was from 1.3 to 11.7 mL/min, and this made a substantial difference in the duration of contact time; around 20–25 min, at the low flow rate (depending on the wash quantity) compared to 2–3 min, the wash solvent spent in contact with the cake at the high wash flowrate. This increased duration of contact time allows for a closer approach to the thermodynamic equilibrium to be achieved (potentially allowing the API to approach the saturation solubility level), resulting in more API dissolving in and being removed with the wash solvent.^30^ This can be observed when comparing results obtained from experiment 2 (Figure S7a, Supporting Information) with experiment 9 (Figure S14a, Supporting Information). For both these experiments, acetonitrile is used as a wash solvent; however, much more API is lost during washing in experiment 9 where 10 rpm filtration/washing rate is used, compared to very little API loss observed during washing in experiment 2, at 100 rpm filtration/washing rate. Also, increasing the amount of the wash solvent used with a higher number of washes again results in a larger amount of API being dissolved, without necessarily changing the extent of impurity removal.

### 4.2 Purity

**Figure 9:**
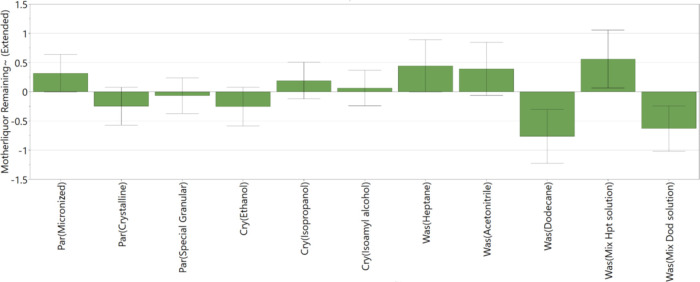
DoE variables that affect mother liquor removal during the wash process; *R*^2^ = 0.77, *Q*^2^ = 0.27, and reproducibility = 0.80.

**Figure 10:**
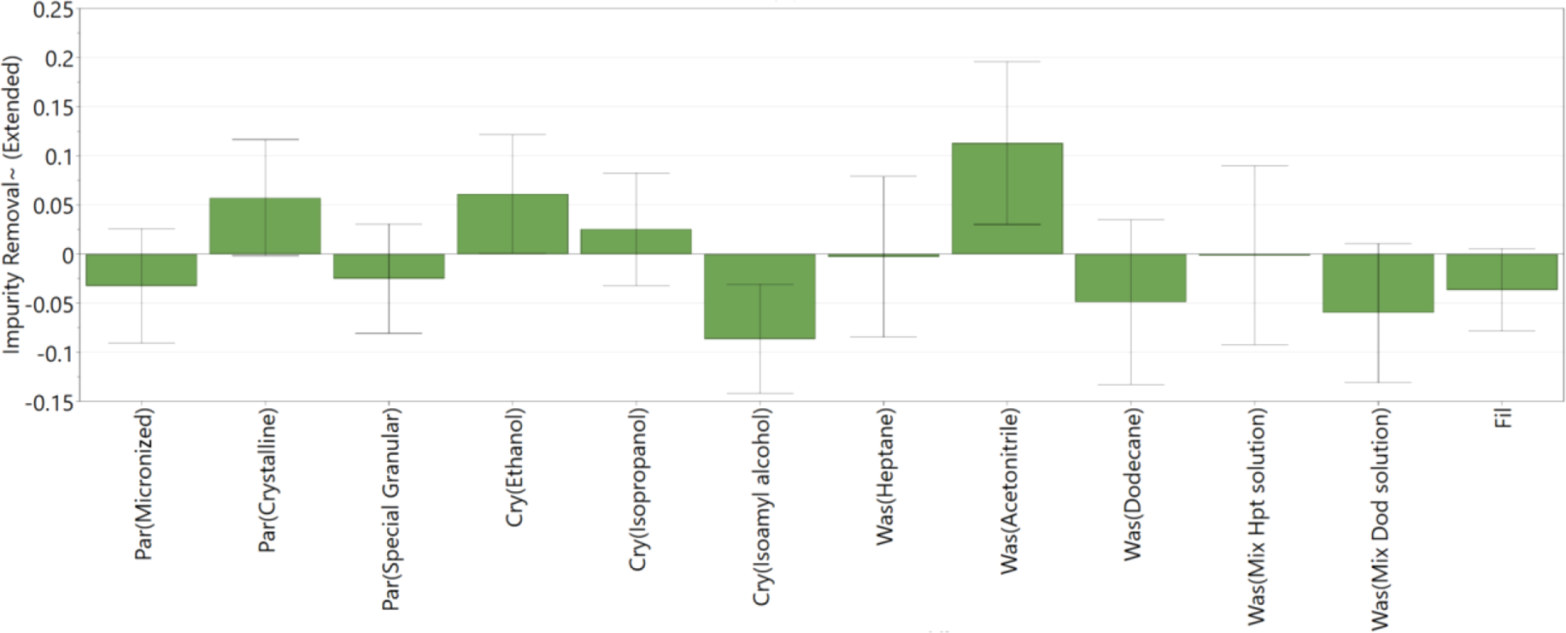
DoE variables that affect blue dye impurity removal during washing; *R*^2^ = 0.79, *Q*^2^ = 0.35, and reproducibility = 0.44.

Figure 9 shows the factors affecting the removal of the mother liquor/crystallization solvent from the filtered API cake during the wash process. The coefficient plot (Figure 9) shows a good reproducibility value and indicates a good fit between the model and the data; however, it does not demonstrate that the model has a good capability to predict responses based on the *Q*^2^ value of 0.27 compared with 0.61 for the prediction of API losses. Figure 10 shows the main factors affecting the removal of the blue dye impurity during the washing process. The coefficient plot (Figure 10) shows good fit between the data and the model and the model’s capability to predict responses. The low reproducibility of the model, 0.44, is due to the variation in the results obtained from the three DoE center point experiments.

The PCM grade, the identity of crystallization and the wash solvent, and the filtration rate are the main factors affecting the removal of mother liquor and blue dye impurity during the washing process (Figures 9 and 10). The increased amount of mother liquor present in the micronized PCM grade, following washing, is consistent with the porosity and tortuosity of the cake. Higher cake tortuosity increases the propensity to trap impure mother liquor in the cake during filtration and so decreases the capability of the washing process to remove the impure mother liquor.^12,20^ Crystalline and granular API grade with a larger crystal size results in larger interstices in the filtered cake, allowing the wash solvent to more easily flow through and penetrate through the whole API particle bed and hence displace the mother liquor which is present.^12,31^

As stated in previous research, the viscosity of the crystallization solvent and the wash solvent should be similar to promote good displacement washing.^31^Figure 9 shows this to be the case with the combination of ethanol as the crystallization solvent and *n*-dodecane as the wash solvent, resulting in the best removal of mother liquor solution as these two solvents have the most similar viscosities to each other (see Supporting Information for the viscosity data of solvents used in study). Isoamyl alcohol was found to be the most difficult crystallization solvent to displace because of its high viscosity, which makes it difficult to displace this mother liquor from the small capillaries in the API cake.

**Figure 11:**
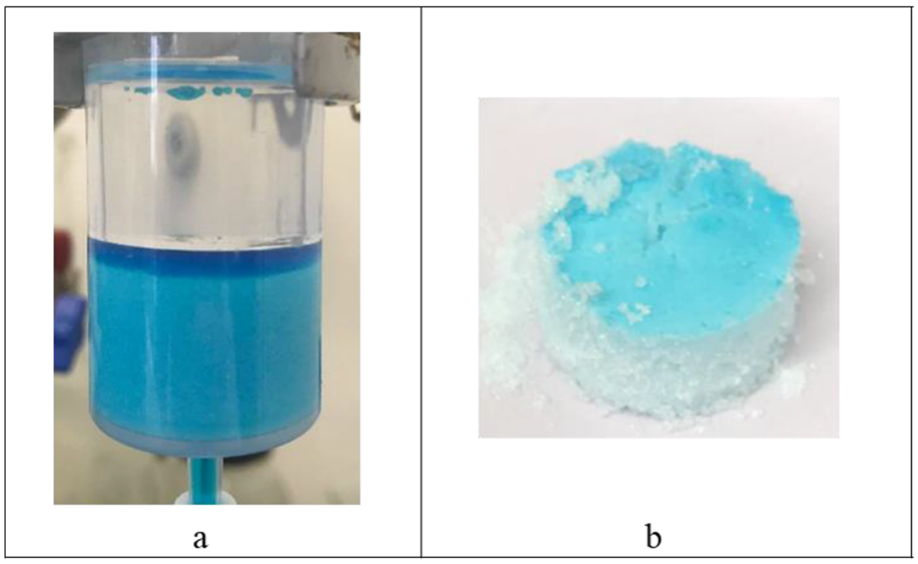
(a) Biotage filter tube from experiment 3 after *n*-dodecane wash solvent addition. (b) PCM API cake obtained at the end of experiment 3 with a layer of the blue dye at the top of the washed cake.

Blue dye impurity removal analysis is carried out by analyzing how quickly the blue dye is removed from the API cake, as explained in Section 3.4 Liquid Filtrate Offline Postanalysis, and also by checking that no blue dye impurity is present inside the washed API cake at the end, as explained in Section 3.5 Solid API Cake Offline Postanalysis. From Figure 10, *n*-dodecane is generally found to be the best wash solvent for the removal of the blue dye impurity. However, when using pure *n*-dodecane as the wash solvent, on some occasions, some of the blue dye impurity and the mother liquor was seen to rise to the top of the filter tube as a layer resting above the added *n*-dodecane wash soon as the wash solvent was added. This can be seen in Figure 11a for experiment 3, where there is a small amount of blue dye which can be seen on the top of the *n*-dodecane wash solvent (experiment 3 is carried out with ethanol as the crystallization solvent and *n*-dodecane as the wash solvent, with one wash using 3 cake volumes at 100 rpm). This could be due to *n*-dodecane being immiscible in the ethanol crystallization solvent and a portion of the mother liquor being disturbed from the surface of the wet filter cake during the wash addition and remaining suspended on the top of the layer of the *n*-dodecane wash solvent. Because there was only 1 wash applied in experiment 3, this blue dye was deposited in the form of a layer at the top of the cake, Figure 11b. When the washed cake from experiment 3 was suspended in the solution to analyze by UV–vis spectroscopy, there was no blue dye retained in the washed cake, and hence, no blue dye was detected, the concentration being below the detection limit and the cake being effectively impurity-free. For experiments using pure *n*-dodecane as the wash solvent, where more than 1 wash was carried out, there was no evidence of a blue dye layer at the top of the cake, indicating that the second and third washes are effective in removing any impurity layer deposited at the top of the cake in the first wash. This phenomenon is not observed when a mixture of *n*-dodecane and the crystallization solvent is used as the first wash solution. (The full list of experiment with different parameters used is provided in Supporting Information, Figure S3.)

In the design space investigated experimentally in the DoE, acetonitrile was found to be the worst wash solvent to use in terms of removal of residual mother liquor solution and the blue dye impurity (Figures 9 and 10). Several factors are believed to contribute to this; acetonitrile has a large viscosity difference compared to the crystallization solvents used, which results in poor displacement washing, (Table S1 in the Supporting Information provides viscosity data for all the solvents used in study). The high solubility of blue dye in acetonitrile relative to the other wash solvents may play a part through the back diffusion of the dye present in mother liquor filling the voids in the API cake and so could contribute to the requirement of a higher volume of the wash solvent for complete washing. Furthermore, the solubility of the API is around one order of magnitude higher in acetonitrile compared to other wash solvents (Table 2), which results in increased API loss during washing.

Of all the experiments undertaken, experiments 9 and 14 were the only ones found to have blue dye still present in the collected filtrate at the end of the experiment; the wash profile curve for both experiments did not reach 0 for the concentration of blue dye (see the Supporting Information). Acetonitrile was the wash solvent used in both experiments, and it shows the highest solubility of both the API and the blue dye impurity (Table 2); the DoE specified one wash of the PCM cake with acetonitrile at a pumping rate of 10 rpm (1.3 mL/min) in both cases. This low flowrate of the solvent through the cake combined with the relatively high solubility of the blue dye in acetonitrile suggests that there is a greater risk of contamination of the wash solvent with the dye because of back mixing. This resulted in less-efficient washing. This, in combination with a small wash volume as required in the DoE, led to inadequate washing in both experiments; consequently, some of the blue dye impurity was still present within the API cake and the wash filtrate at the end of the washing process.^24^

As mentioned previously, the filtration rate is important during washing as it plays a role in minimizing back mixing of impurity or mother liquor with the wash solvent. The use of a pumping rate of 55 rpm with granular PCM grade in the DoE midpoint experiments (experiments 20, 21, and 22) was found to be the best flow rate for removing both the colored impurity and the crystallization solvent. The is evident from the ^1^H-NMR results of the samples taken from the washed cakes that show that the DoE midpoint experiments have the least amount of mother liquor present at the end of washing (see the Supporting Information). This is believed to be mainly due to the time allowed for the wash solvent to flow through the cake to achieve complete removal of impurity and mother liquor without causing any back mixing.

### 4.3 Particle Size Distribution

Conducting particle size analysis of the washed API product before it is dried enables differentiation between agglomeration caused by the washing process and those agglomerates that are formed and are further strengthened by drying. Laser diffraction measurement was found to be a fairly effective way of observing any change in the PSD of the API caused by filtration and washing.

**Figure 12:**
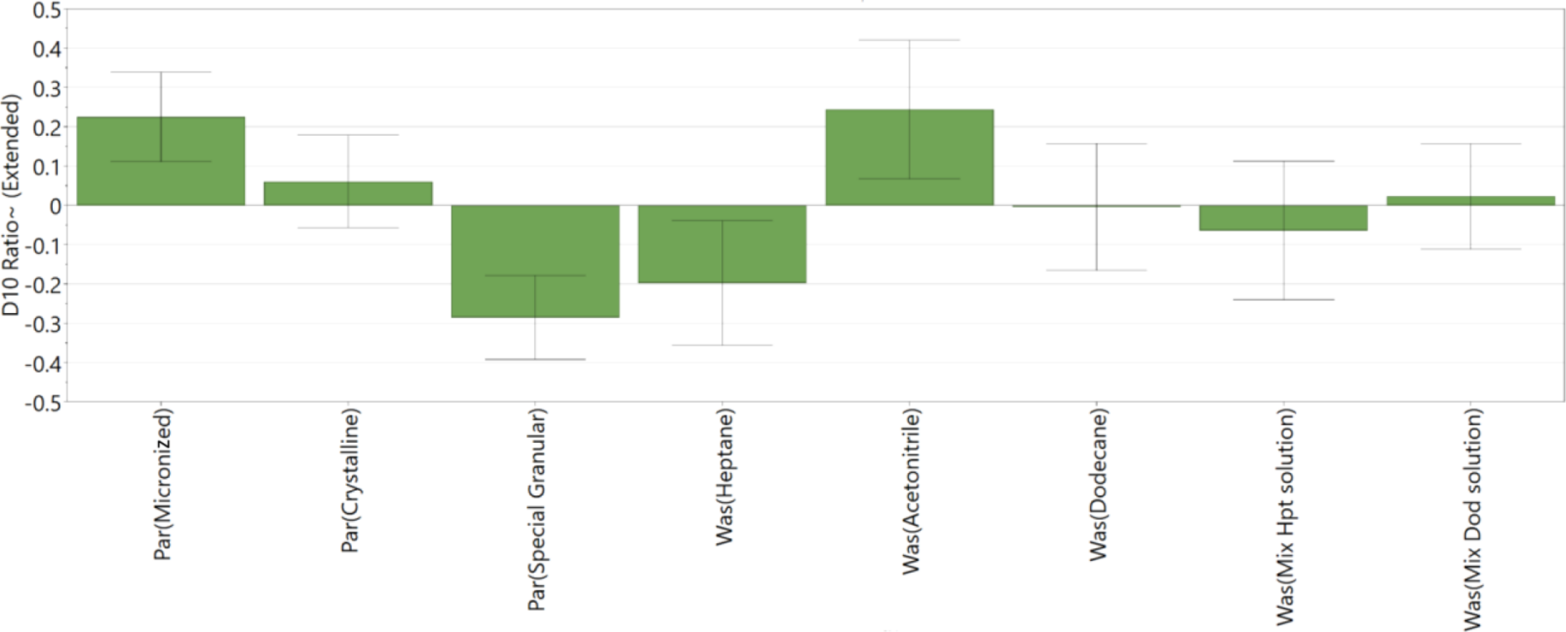
DoE variables that affect the PSD (*D*_10_) of washed cake — change in *D*_10_; *R*^2^ = 0.80, *Q*^2^ = 0.52, and reproducibility = 0.97.

**Figure 13:**
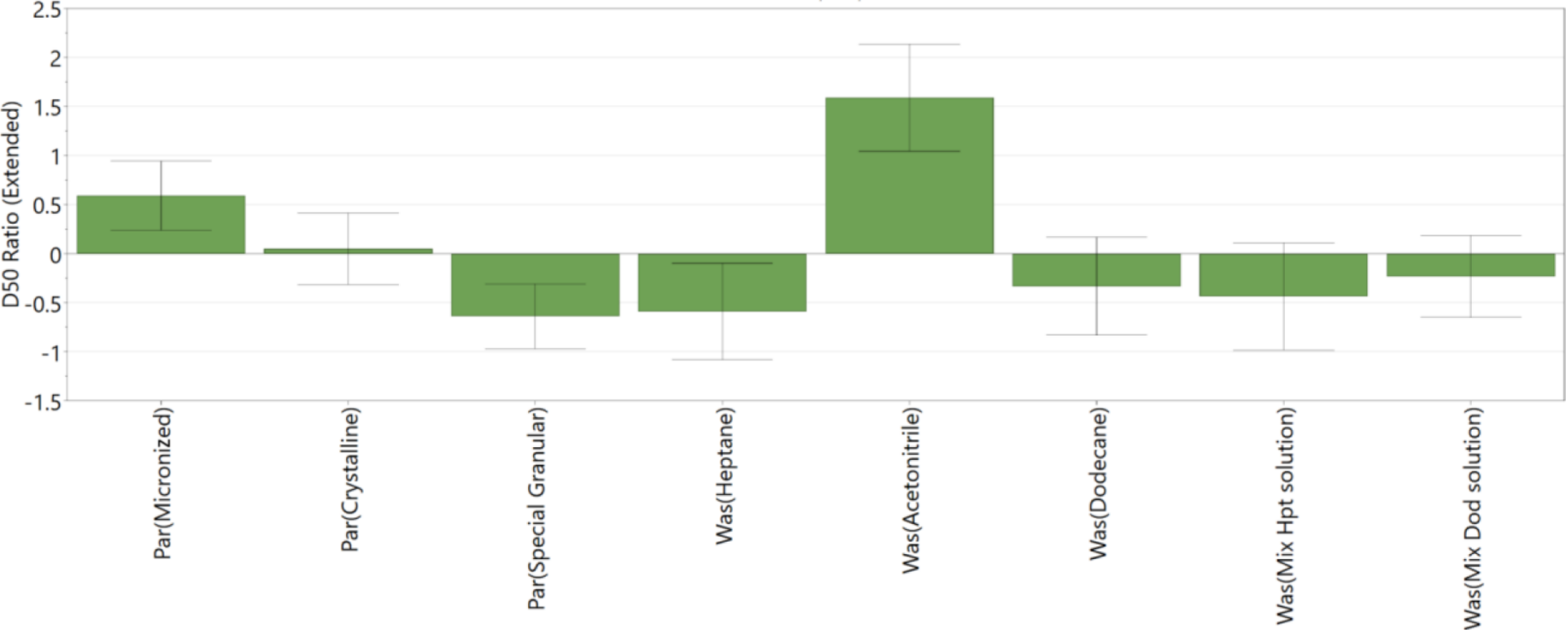
DoE variables that affect PSD (*D*_50_) of washed cake — change in *D*_50_; *R*^2^ = 0.82, *Q*^2^ = 0.58, and reproducibility = 0.97.

**Figure 14:**
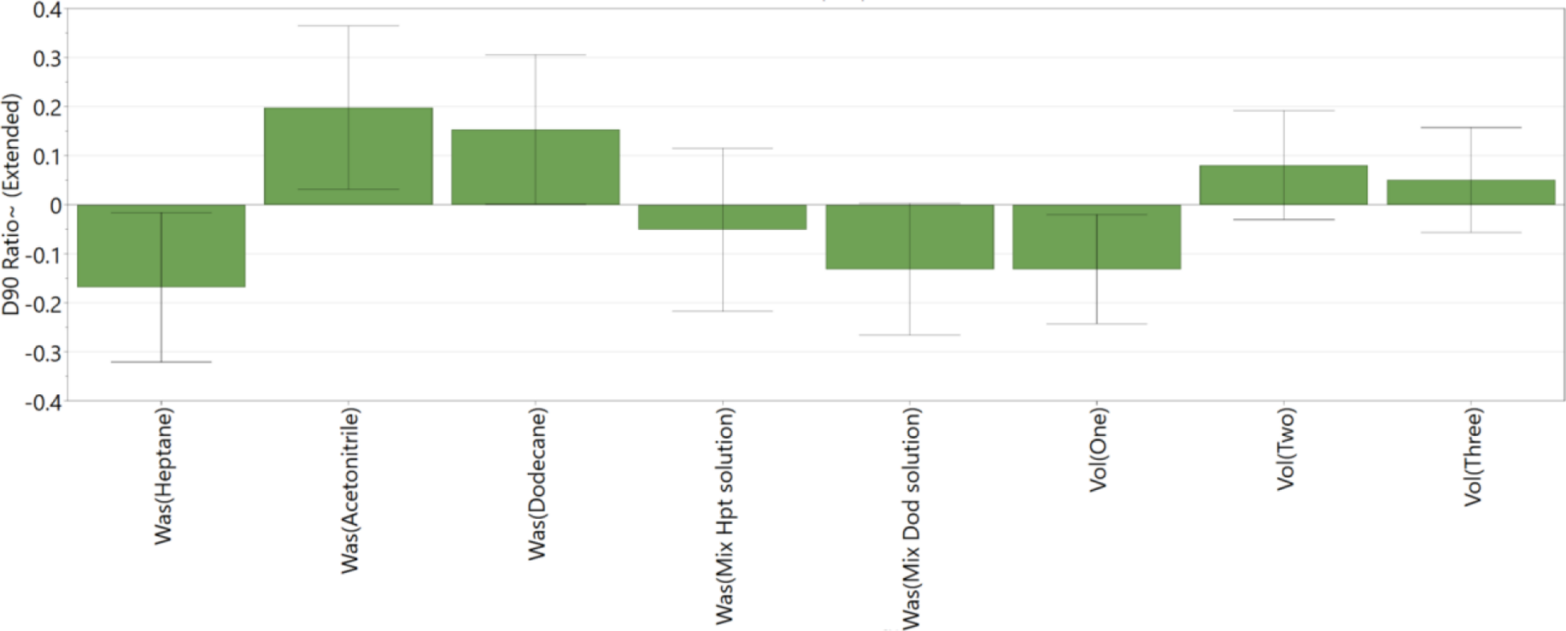
DoE variables that affect PSD (*D*_90_) of washed cake — change in *D*_90_; *R*^2^ = 0.60, *Q*^2^ = 0.05, and reproducibility = 0.81.

Figures 12, 13, and 14 show the main factors affecting the PSD *D*_10_, *D*_50_, and *D*_90_ during the washing process. All three coefficient plots (Figures 12, 13, and 14) show good reproducibility values. Coefficient plots for *D*_10_ and *D*_50_ (Figures 12 and 13, respectively) show good fit between the data and the model demonstrating the model’s capability to predict responses. The coefficient plot for *D*_90_ (Figure 14) shows good fit between the model and the data; however, the low reproducibility value indicates that the model has limited usefulness in predicting responses.

The main factors linked to PSD change during the washing process are the PCM API grade and the wash solvent identity. Processing micronized PCM results in an increase in the PSD during washing, and this is probably due to the wide PSD of the raw material, a contributing factor may be the finer particles being located in the small voids within the cake acting as the bridge formation agents, correlated to the high surface area present.^29,32^ Granular PCM tended to maintain the initial PSD after the washing process.

Acetonitrile was found to be the worst wash solvent in terms of causing agglomeration, leading to an increasing PSD, and this is consistent with the findings that acetonitrile is the worst wash solvent in this study in terms of the removal of mother liquor and impurities due to back mixing and solvent viscosity differences, see Section 3.3. Using acetonitrile as the wash solvent would result in a final washed cake with the saturated solvent in the porous cake with API dissolved with it. Hence, the presence of saturated mother liquor solution or solvent with API dissolved in it at the end of the washing process could result in the API being deposited during the drying stage and causing severe agglomeration. The wet dispersion particle size analysis employed isooctane as the dispersant, in which the API has negligible solubility. The presence of saturated acetonitrile as a solvent residue in the washed cake could potentially result in an antisolvent effect. As the wet cake sample is dispersed, the acetonitrile interaction with isooctane would lead to the deposition of API adversely affecting the PSD analysis of the sample.

**Figure 15:**
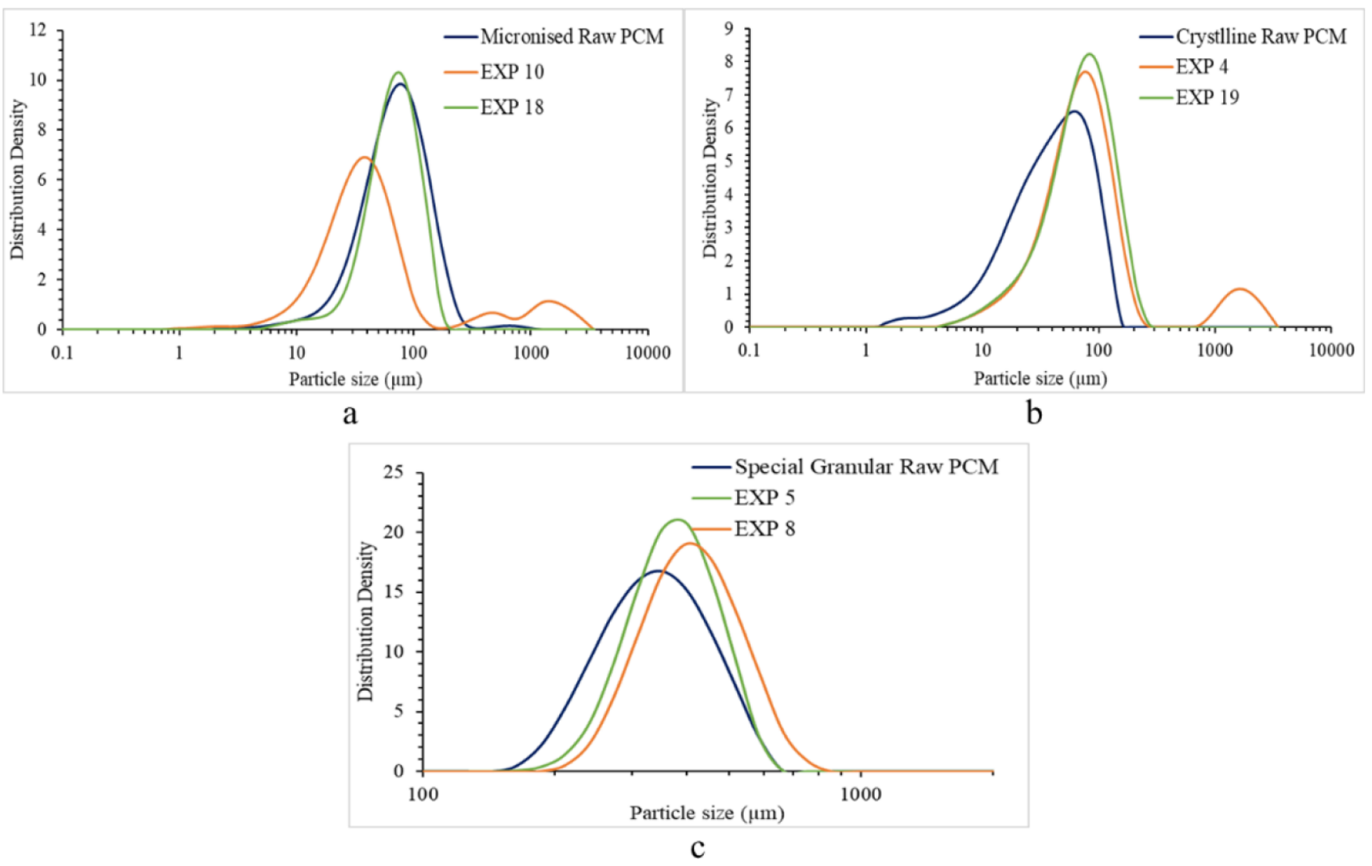
(a) PSD of raw micronized PCM and of final washed cakes from EXP 10 (*D*_10_: 15.2 μm, *D*_50_: 43.8 μm, *D*_90_: 638.3 μm) and EXP 18 (*D*_10_: 35.1 μm, *D*_50_: 72.9 μm, *D*_90_: 128.2 μm). (b) PSD of raw crystalline PCM and of final washed cakes from EXP 4 (*D*_10_: 27.8 μm, *D*_50_: 76.5 μm, *D*_90_: 201.0 μm) and 19 (*D*_10_: 26.8 μm, *D*_50_: 76.7 μm, *D*_90_: 154.4 μm). (c) PSD of raw granular PCM and of final washed cakes from EXP 5 (*D*_10_: 292.4 μm, *D*_50_: 397.4 μm, *D*_90_: 531.8 μm) and EXP 8(*D*_10_: 312.8 μm, *D*_50_: 437.0 μm, *D*_90_: 606.4 μm).

The use of the wash solvent in which the API has low solubility, such as *n*-heptane and *n*-dodecane, carries the risk of precipitation of API during washing because of the antisolvent effect occurring as the wash solvent interacts with the saturated mother liquor occupying the voids in the cake, potentially a cause of agglomeration.^22^Figure 15 gives an example of how using a mixture of the wash solvent and the crystallization solvent as the initial wash solution reduces this antisolvent effect and hence lowers the extent of agglomeration during washing. Figure 15 shows the PSD obtained for the three different PCM grades used within this study as well as the PSD of some of the washed cake samples from some of the experiments. Table 4 contains the factors and washing regime used in all the experiments presented in Figure 15.

**Table 4:** Factors Used for Experiments Presented in Figure 15

	micronized		crystalline		granular	
	EXP 10	EXP 18	EXP 4	EXP 19	EXP 5	EXP 8
crystallization solvent	ethanol	ethanol	isopropanol	isopropanol	isoamyl alcohol	isoamyl alcohol
wash solvent	dodecane	mix dodecane sol	dodecane	mix dodecane sol	mix N-heptane sol	N-heptane
filtration rate in rpm (in mL/min)	100 (11.7)	10 (1.3)	10 (1.3)	100 (11.7)	10 (1.3)	100 (11.7)
volume of wash solvent (void volume)	2	3	1	3	1	2
no. of washes	3	2	1	3	3	1

As shown in Figure 15a, micronized PCM and ethanol are used in all the experiments. As shown in Figure 15b, crystalline PCM and isopropanol are used in all the experiments, and as shown in Figure 15c, granular PCM and isoamyl alcohol are used in all the experiments. All the experiments showed an increase in the PSD of the washed crystal particles, from the initial raw material. A *D*_10_, *D*_50_, and *D*_90_ comparison of the raw crystal particles with the washed cake showed an increase of between 1.9 and 24 times the *D*_10_, *D*_50_, and *D*_90_ of the original material when washed with a pure wash solvent. An increase in *D*_10_ is believed to be mainly due to the antisolvent effect within the cake structure, causing precipitation when the wash solvent in which the API and impurity have the lowest solubility comes in contact with the supersaturated crystallization solvent present within the cake structure, causing an increase in supersaturation. This results in either further precipitation within the cake bed or crystal bridges being formed, causing agglomeration, which is evident with increase in both *D*_50_ and *D*_90_.^22^

Employing a more sophisticated wash strategy, in the case of all three crystallization solvents, where the first wash is carried out using a mixture of crystallization and the wash solvent, followed by further washing using the pure wash solvent, as employed in experiments in 5, 18, and 19 (Figure 15) produces much less agglomeration and precipitation caused by the antisolvent effect. The *D*_10_, *D*_50_, and *D*_90_ in this case has only increased between 1.1 and 2.6 times the *D*_10_, *D*_50_, and *D*_90_ of the raw material.

## 5 Conclusions

Washing API crystals is an important part of the isolation process to deliver a crystalline product of the desired purity, PSD, and yield. The constant rate filtration/washing methodology developed in this study is easily implemented using readily available laboratory equipment and allows detailed investigation of filtrate fractions from the washing process. The analysis of the filtrate has been shown to be useful in determining the endpoint of washing, the amount of API lost during the washing process, and the likely extent of agglomeration occurring during washing to be evaluated. Knowing these properties allows for the development of more sustainable washing processes with less solvent waste and improved product quality.

This work demonstrates that agglomerate formation during isolation starts at the washing stage where inappropriate solvent choices combined with a poorly designed washing strategy can lead to the formation of agglomerates driven by the retention of the crystallization solvent in the wet filter cake. This then results in further strengthening and formation of larger agglomerates during drying, necessitating additional steps such as milling which increases production time and cost. The use of laser diffraction for particle size analysis of the damp-washed cake was found to be satisfactory in identifying agglomerate formation during washing. However further work is required to optimize the method for particle size analysis of washed cakes as well as developing new approaches to allow for inline analysis of various parameters during the washing process, making it more efficient and economical.

The ideal case for achieving good washing was found to be when the starting point was crystalline PCM wet cake fully saturated in ethanol. Even though *n*-dodecane was found to be the best wash solvent in terms of performing displacement washing, the immiscibility of the wash solvent with the crystallization solvent was found to jeopardize the removal of the blue dye impurity. In addition, the high boiling point of *n*-dodecane makes it difficult to remove during the drying process. Using lower flowrates resulted in the back diffusion of the crystallization solvent, while using larger amounts of the wash solvent was found to be inefficient because of increased API loss and wash solvent being consumed after the point at which all the impurity has been removed. Whilst these results are specific to the PCM samples studied, for another API, the same principles would be applied to develop a washing regime tailored to the specific API but along the same lines as described above.

The constant rate washing methodology developed using the blue dye impurity has been found to be very effective in analyzing washing processes and designing a washing strategy. A future investigation will involve using API with structurally related impurities and designing a constant rate filtration strategy to demonstrate how this approach could be implemented on an industrial scale to washing processes.

## Supporting Information Available

Table S1: Main properties of the solvents used in this work; Figure S1: Factors of DoE; Figure S2: Responses of DoE; Figure S3: DoE experimental worksheet with factors; Figure S4: DoE experimental worksheet with responses; Figure S5: Images of all the API washed cakes taken at the end of the experiment and sorted in terms of wash solvent, number of washes, and void volume of wash solvent; Figure S6–S27: Results obtained from experiments 1–22 (PDF)

## Abbreviations

API: active pharmaceutical ingredient
PCM: paracetamol
PSD: particle size distribution
^1^H NMR: proton nuclear magnetic resonance
DoE: design of experiment
