# Supplementary material for: Employing Constant Rate Filtration To Assess Active Pharmaceutical Ingredient Washing Efficiency
Source: Org Process Res Dev. 2021 Dec 21;26(1):97–110. doi: 10.1021/acs.oprd.1c00272 (PMC8787817; doi:10.1021/acs.oprd.1c00272)
Supplement: Supplementary file 1 — op1c00272_si_001.pdf [file op1c00272_si_001.pdf]

Supporting Information

Employing constant rate filtration to assess active pharmaceutical ingredient (API) washing efficiency

Muhid Shahid[*1], *Chloé Faure*[2], *Sara Ottoboni*[1], *Leo Lue*[3], *Chris Price*[1,3]

[1] *EPSRC Continuous Manufacturing & Advanced Crystallisation (CMAC) Future Manufacturing Research Hub, University of Strathclyde, Glasgow, G1 1RD, UK*

2 Département de Genie Chimique-Génie des Procédés, UT Paul Sabatier, 137 Avenue de Rangueil, BP 67701, 31077 Toulouse, Cedex 4 France

[3] *Department of Chemical and Process Engineering, University of Strathclyde, Glasgow, G1 1XJ, UK*

*****Email: muhid.shahid@gmail.com

Table S1: Main properties of the solvents used in this work.[1-5]

Solvent	Boiling point (ºC)	Enthalpy of vaporization (kJ/mol)	Viscosity (cP) (Temperature ºC)	Density (g/ml) (Temperature ºC)	Surface tension (mN/m) (Temperature ºC)
Ethanol	78.4	38.58	1.26 (20)	0.79 (20)	21.99 (20)
Isopropanol	82.2	39.85	2.1 (25)	0.78 (25)	21.4 (20)
Isoamyl alcohol	132	55.2	3.74 (25)	0.81 (15)	24.77 (15)
Acetonitrile	81.6	33.23	0.35 (20)	0.78 (20)	29.04 (20)_
n-Heptane	98.4	31.77	0.397 (25)	0.68 (20)	19.7 (20)
n-Dodecane	216.3	61.52	1.5 (25)	0.75 (20)	25.35 (20)

Factors

	Name	Abbr.	Units	Type	Settings	Transform	Precision
1	**Paracetamol Grade**	Par		Qualitative	Micronised, Crystalline, Special Granular		
2	**Crystallisation solvent**	Cry		Qualitative	Ethanol, Isopropanol, Isoamyl alcohol		
3	**Wash Solvent**	Was		Qualitative	Heptane, Acetonitrile, Dodecane, Mix Hpt solution, Mix Dod solution		
4	**Filtration & Washing Rate**	Fil	rpm	Quantitative	10 to 100	None	2.25
5	**Volume of Wash Solvent**	Vol		Qualitative	One, Two, Three		
6	**Number of washes**	Num		Quantitative	1 to 3	None	0.05
		Double-click here	*to add*	*a new factor*			

Figure S1: Factors of DoE

Responses

	Name	Abbr.	Units	Transform	Type	Min	Target	Max
1	**Impurity Removal**	IR		Log: 10Log(Y)	Regular		1	
2	**Motherliquor Remaining**	MLR		Log: 10Log(Y)	Regular			
3	**API Lost to Washing**	APIL	g	Log: 10Log(Y+0.1)	Regular			
4	**D10 Ratio**	D10		Log: 10Log(Y)	Regular			
5	**D50 Ratio**	D50		None	Regular			
6	**D90 Ratio**	D90		Log: 10Log(Y)	Regular			
		Double-click here	*to add*	*a new response*				

Figure S2: Responses of DoE

Worksheet

	1	2	3	4	5	6	7	8	9	10
	Exp No	Exp Name	Run Order	Incl/Excl	Paracetamol Grade	Crystallisation solvent	Wash Solvent	Filtration & Washing Rate	Volume of Wash Solvent	Number of washes
1	1	N1	17	Incl	Crystalline	Ethanol	Heptane	10	One	3
2	2	N2	22	Incl	Micronised	Isopropanol	Acetonitrile	100	One	1
3	3	N3	5	Incl	Special Granular	Ethanol	Dodecane	100	One	3
4	4	N4	4	Incl	Crystalline	Isopropanol	Dodecane	10	One	1
5	5	N5	14	Incl	Special Granular	Isoamyl alcohol	Mix Hpt solution	10	One	3
6	6	N6	10	Incl	Micronised	Isoamyl alcohol	Mix Dod solution	100	One	2
7	7	N7	13	Incl	Micronised	Isoamyl alcohol	Heptane	10	Two	3
8	8	N8	15	Incl	Special Granular	Isoamyl alcohol	Heptane	100	Two	1
9	9	N9	1	Incl	Crystalline	Isopropanol	Acetonitrile	10	Two	1
10	10	N10	20	Incl	Micronised	Ethanol	Dodecane	100	Two	3
11	11	N11	9	Incl	Crystalline	Ethanol	Mix Hpt solution	100	Two	2
12	12	N12	7	Incl	Special Granular	Isopropanol	Mix Dod solution	10	Two	3
13	13	N13	21	Incl	Special Granular	Isopropanol	Heptane	100	Three	1
14	14	N14	3	Incl	Special Granular	Ethanol	Acetonitrile	10	Three	1
15	15	N15	18	Incl	Crystalline	Isoamyl alcohol	Acetonitrile	100	Three	3
16	16	N16	16	Incl	Crystalline	Isoamyl alcohol	Dodecane	10	Three	1
17	17	N17	2	Incl	Micronised	Isopropanol	Mix Hpt solution	10	Three	3
18	18	N18	8	Incl	Micronised	Ethanol	Mix Dod solution	10	Three	2
19	19	N19	12	Incl	Crystalline	Isopropanol	Mix Dod solution	100	Three	3
20	20	N20	6	Incl	Special Granular	Isoamyl alcohol	Mix Dod solution	55	Three	2
21	21	N21	11	Incl	Special Granular	Isoamyl alcohol	Mix Dod solution	55	Three	2
22	22	N22	19	Incl	Special Granular	Isoamyl alcohol	Mix Dod solution	55	Three	2

Figure S3: DoE experimental worksheet with factors

11	12	13	14	15	16
Impurity Removal	Motherliquor Remaining	API Lost to Washing	D10 Ratio	D50 Ratio	D90 Ratio
6	0.0317	0	2.11	1.758	0.743
5	0.5148	0.66	7.71	4.456	1.102
4	0.0147	0	1.31	1.207	1.168
5	0.0135	0.07	2.22	1.74	1.984
3	0.5488	0.17	1.18	1.1	1.027
3	0.01	0.39	6.22	3.25	1.507
3	0.5448	0	1.65	1.787	1.47
4	0.2843	0	1.27	1.209	1.171
7	0.1528	0.37	4.83	3.617	2.906
3	0.0042	0	2.33	1.59	3.221
5	0.0281	0.31	1.73	1.354	1.228
4	0.0088	0.17	1.27	1.29	1.331
3	0.0691	0	0.68	1.123	1.088
7	0.021	0	2.12	3.348	4.361
4	0.1135	1.48	9.72	29.436	24.205
4	0.0019	0.08	5.85	3.055	2.204
5	0.3657	0.95	4.61	2.888	1.983
4	0.0603	0	5.939	2.647	0.647
4	0.0207	0.29	2.15	1.745	1.524
2	0.0049	0.1	1.29	1.38	1.539
3	0.0072	0.05	1.29	1.223	1.187
3	0.0214	0.07	1.05	1.038	1.03

Figure S4: DoE experimental worksheet with responses

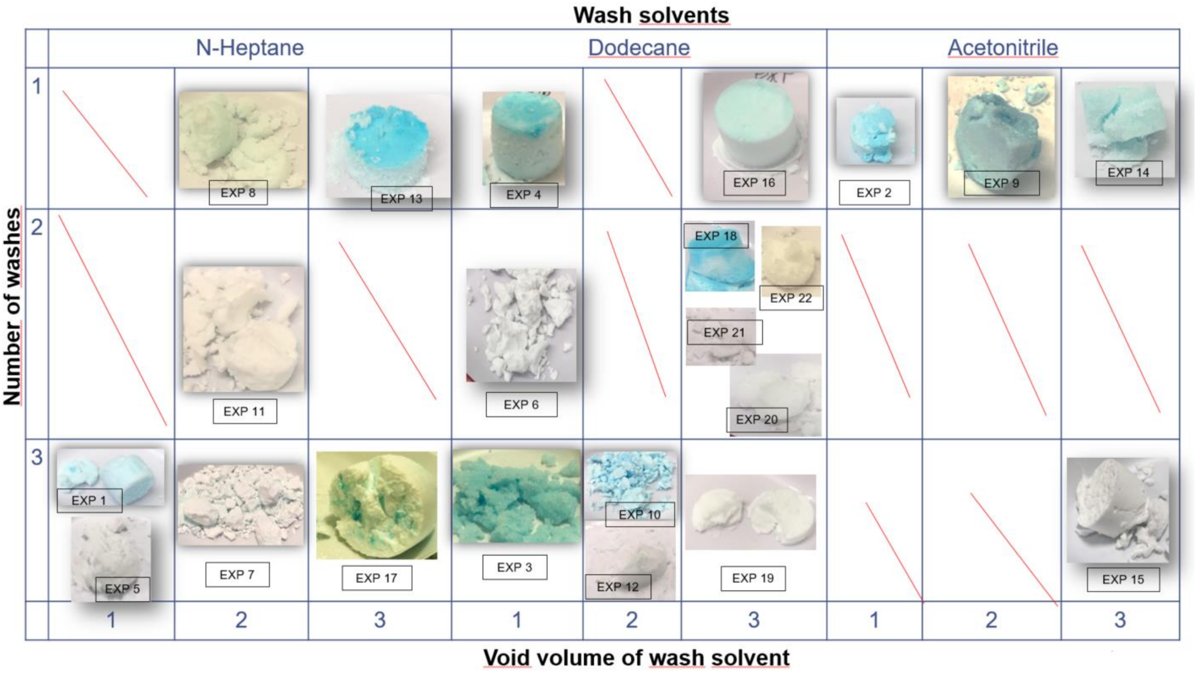

Figure S5: Images of all the API washed cakes taken at the end of experiment and sorted in terms of wash solvent, number of washes and void volume of wash solvent

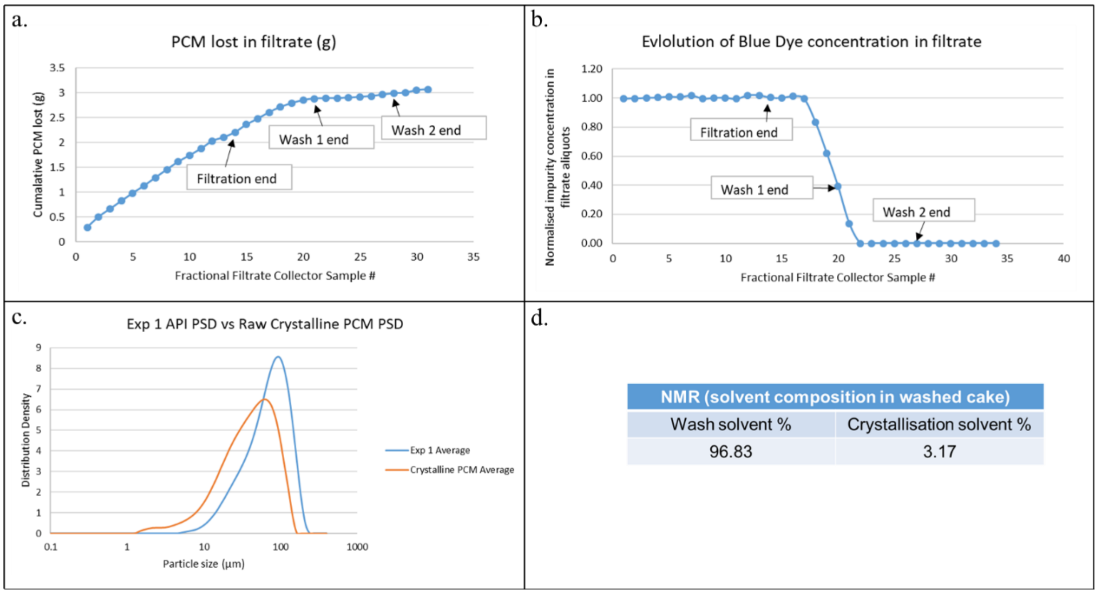

NMR (solvent composition in washed cake)	
Wash solvent %	Crystallisation solvent %
96.83	3.17

Figure S6: Results obtained from experiment 1; crystallisation solvent: ethanol; wash solvent: n-heptane; API grade: crystalline; filtration rate (rpm): 10; volume of wash: 1 void volumes; number of washes: 3. a.) Graph showing cumulative API loss in filtrate samples throughout the experiment, mass of PCM API lost during wash = 1.48 g. b.) Normalised concentration of blue dye impurity in each filtrate sample obtained throughout the experiment. c.) Particle size distribution of the raw paracetamol API and the washed cake sample obtained at the end of the washing experiment, from the damp cake. d.) ^{1}H-NMR analysis results showing the residual crystallisation solvent content in the final washed cake.

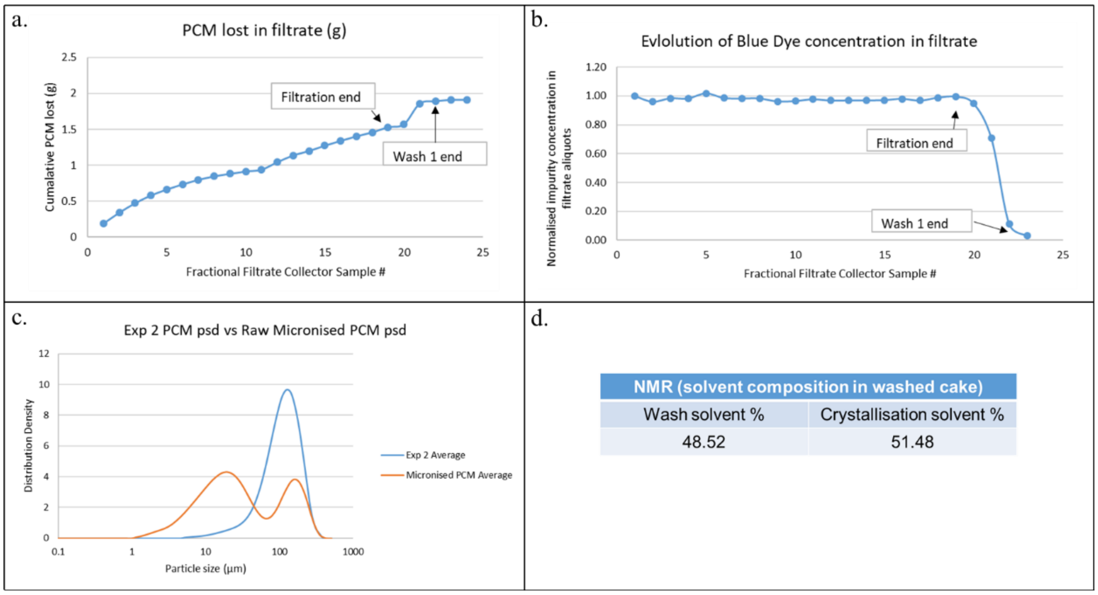

NMR (solvent composition in washed cake)	
Wash solvent %	Crystallisation solvent %
48.52	51.48

Figure S7: Results obtained from experiment 2; crystallisation solvent: isopropanol; wash solvent: acetonitrile; API grade: micronised; filtration rate (rpm): 100; volume of wash: 1 void volumes; number of washes: 1. a.) Graph showing cumulative API loss in filtrate samples throughout the experiment, mass of PCM API lost during wash = 0.66 g. b.) Normalised concentration of blue dye impurity in each filtrate sample obtained throughout the experiment. c.) Particle size distribution of the raw paracetamol API and the washed cake sample obtained at the end of the washing experiment, from the damp cake. d.) ^{1}H-NMR analysis results showing the residual crystallisation solvent content in the final washed cake.

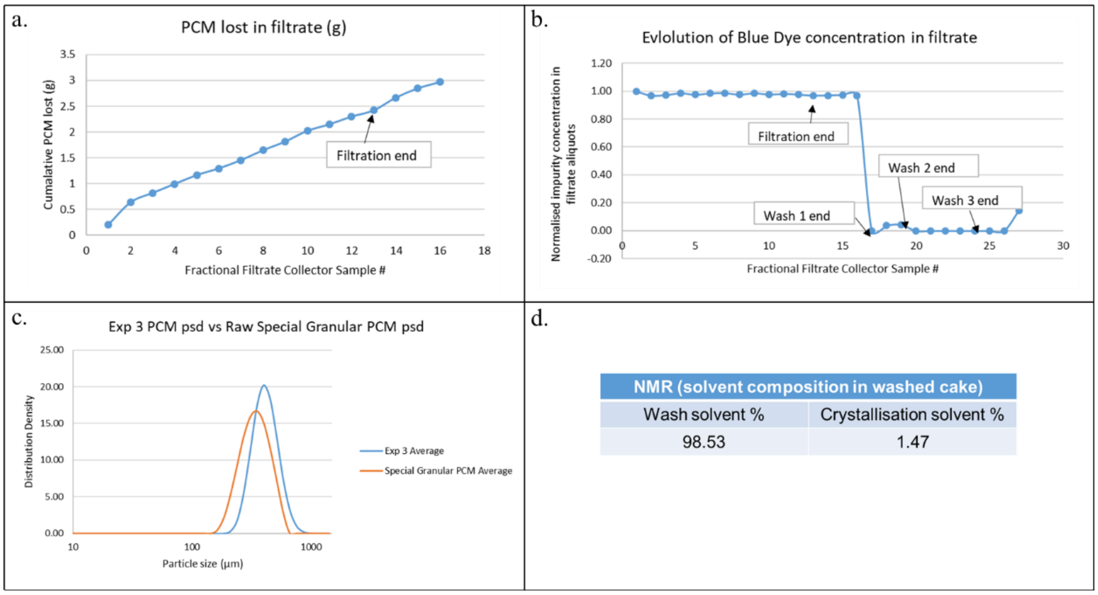

NMR (solvent composition in washed cake)	
Wash solvent %	Crystallisation solvent %
98.53	1.47

Figure S8: Results obtained from experiment 3; crystallisation solvent: ethanol; wash solvent: n-dodecane; API grade: special granular; filtration rate (rpm): 100; volume of wash: 1 void volumes; number of washes: 3. a.) Graph showing cumulative API loss in filtrate samples throughout the experiment, mass of PCM API lost during wash = 0 g. b.) Normalised concentration of blue dye impurity in each filtrate sample obtained throughout the experiment. c.) Particle size distribution of the raw paracetamol API and the washed cake sample obtained at the end of the washing experiment, from the damp cake. d.) ^{1}H-NMR analysis results showing the residual crystallisation solvent content in the final washed cake.

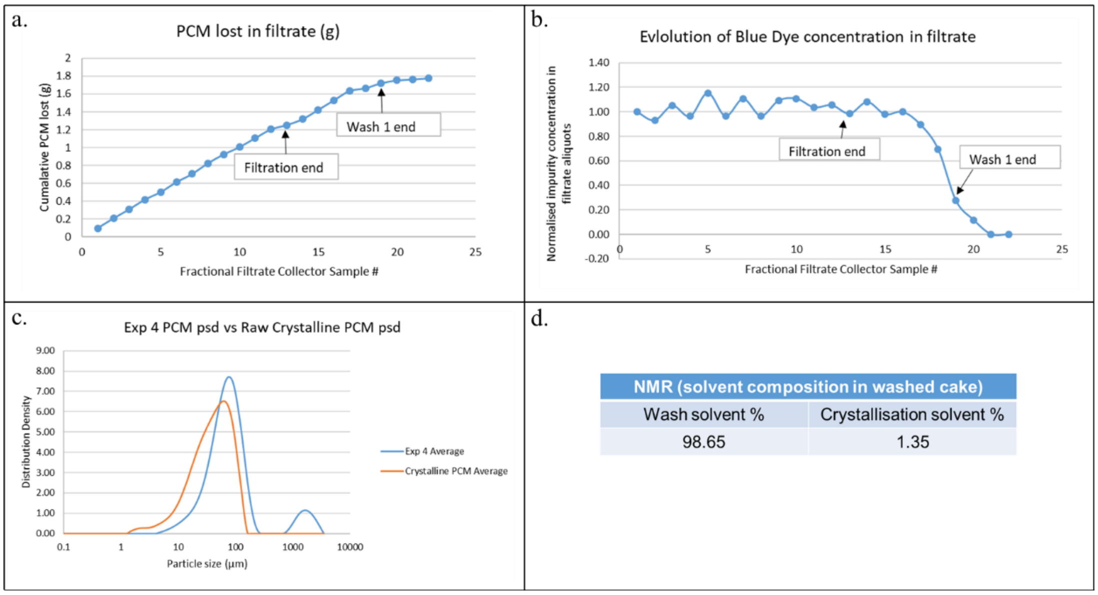

NMR (solvent composition in washed cake)	
Wash solvent %	Crystallisation solvent %
98.65	1.35

Figure S9: Results obtained from experiment 4; crystallisation solvent: isopropanol; wash solvent: n-dodecane; API grade: crystalline; filtration rate (rpm): 10; volume of wash: 1 void volumes; number of washes: 1. a.) Graph showing cumulative API loss in filtrate samples throughout the experiment, mass of PCM API lost during wash = 0.07 g. b.) Normalised concentration of blue dye impurity in each filtrate sample obtained throughout the experiment. c.) Particle size distribution of the raw paracetamol API and the washed cake sample obtained at the end of the washing experiment, from the damp cake. d.) ^{1}H-NMR analysis results showing the residual crystallisation solvent content in the final washed cake.

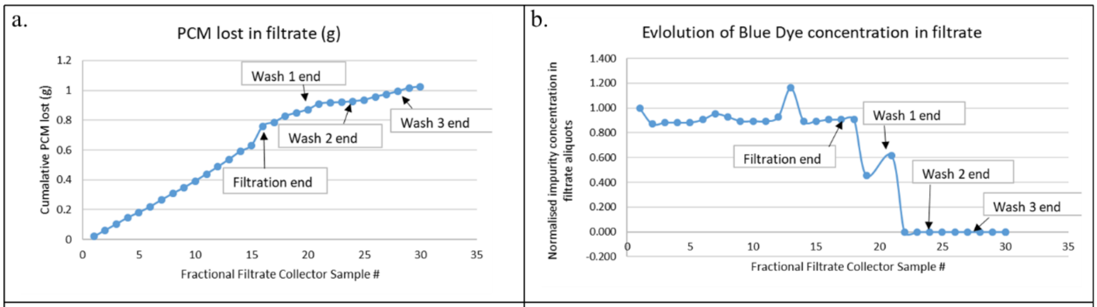

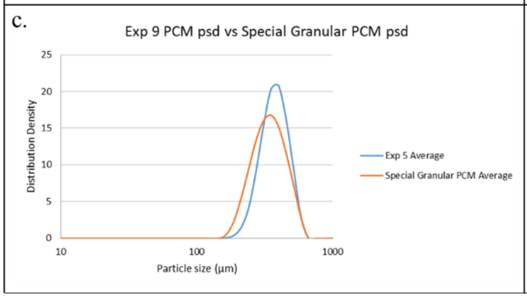

d.

NMR (solvent composition in washed cake)	
Wash solvent %	Crystallisation solvent %
45.12	54.88

Figure S10: Results obtained from experiment 5; crystallisation solvent: isoamyl alcohol; wash solvent: mix n-heptane solution; API grade: special granular; filtration rate (rpm): 10; volume of wash: 1 void volumes; number of washes: 3. a.) Graph showing cumulative API loss in filtrate samples throughout the experiment, mass of PCM API lost during wash = 0.17 g. b.) Normalised concentration of blue dye impurity in each filtrate sample obtained throughout the experiment. c.) Particle size distribution of the raw paracetamol API and the washed cake sample obtained at the end of the washing experiment, from the damp cake. d.) ^{1}H-NMR analysis results showing the residual crystallisation solvent content in the final washed cake.

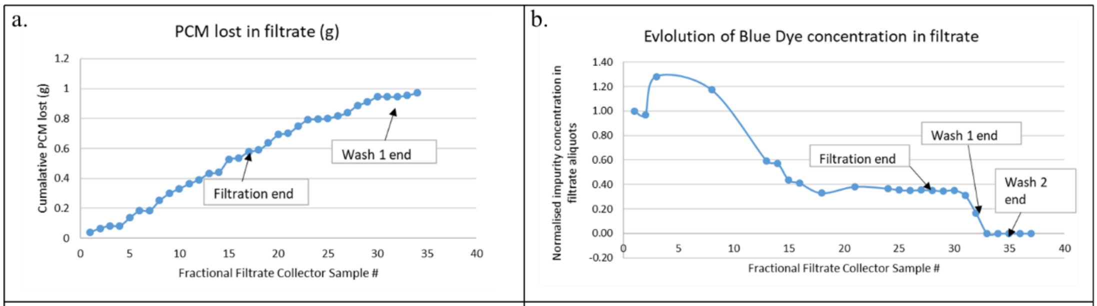

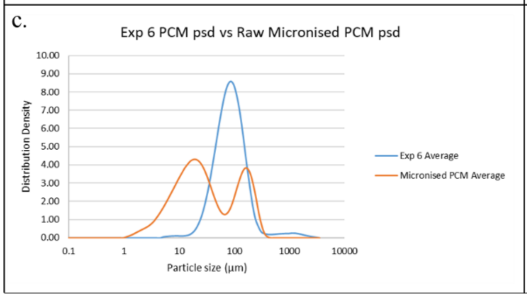

d.

NMR (solvent composition in washed cake)	
Wash solvent %	Crystallisation solvent %
99.00	1.00

Figure S11: Results obtained from experiment 6; crystallisation solvent: isoamyl alcohol; wash solvent: mix n-dodecane solution; API grade: micronised; filtration rate (rpm): 100; volume of wash: 1 void volumes; number of washes: 2. a.) Graph showing cumulative API loss in filtrate samples throughout the experiment mass of PCM API lost during wash = 0.39 g. b.) Normalised concentration of blue dye impurity in each filtrate sample obtained throughout the experiment. c.) Particle size distribution of the raw paracetamol API and the washed cake sample obtained at the end of the washing experiment, from the damp cake. d.) ^{1}H-NMR analysis results showing the residual crystallisation solvent content in the final washed cake.

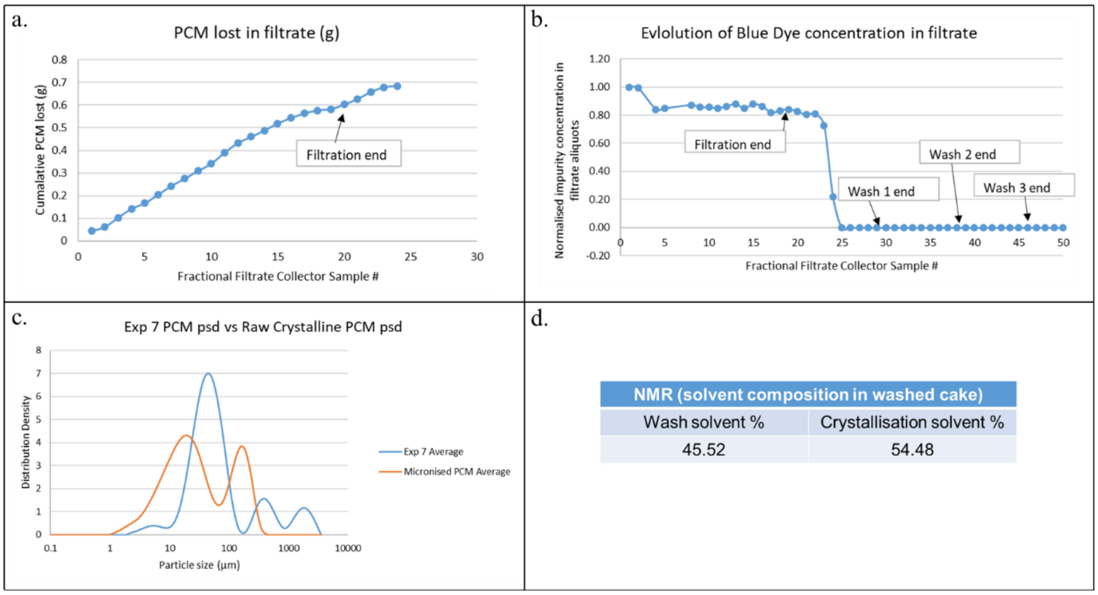

NMR (solvent composition in washed cake)	
Wash solvent %	Crystallisation solvent %
45.52	54.48

Figure S12: Results obtained from experiment 7; crystallisation solvent: isoamyl alcohol; wash solvent: n-heptane; API grade: micronised; filtration rate (rpm): 10; volume of wash: 2 void volumes; number of washes: 3. a.) Graph showing cumulative API loss in filtrate samples throughout the experiment, mass of PCM API lost during wash = 0 g. b.) Normalised concentration of blue dye impurity in each filtrate sample obtained throughout the experiment. c.) Particle size distribution of the raw paracetamol API and the washed cake sample obtained at the end of the washing experiment, from the damp cake. d.) ^{1}H-NMR analysis results showing the residual crystallisation solvent content in the final washed cake.

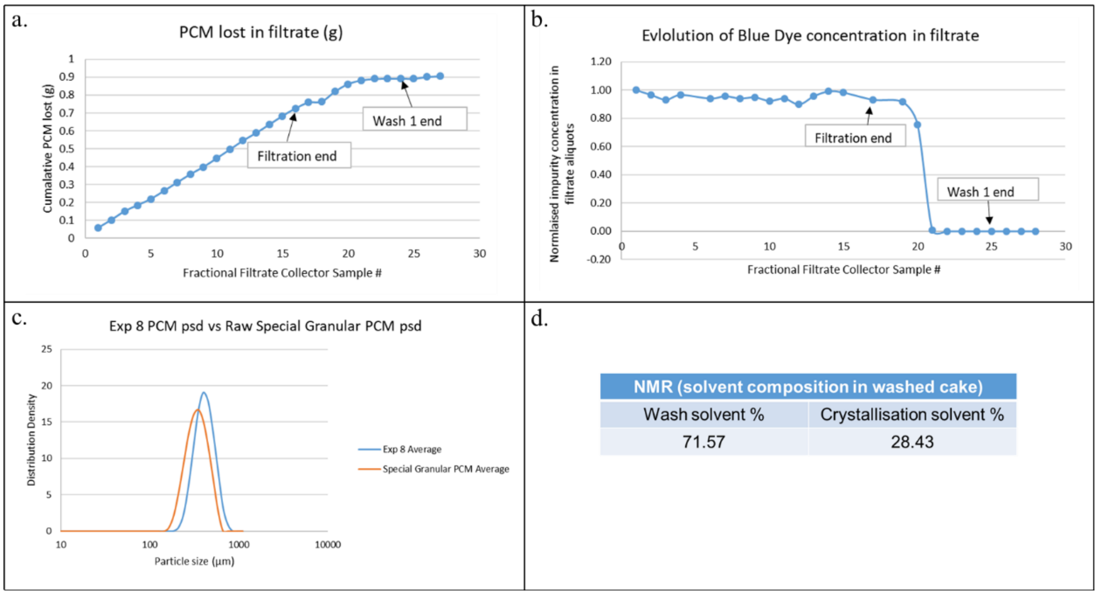

NMR (solvent composition in washed cake)	
Wash solvent %	Crystallisation solvent %
71.57	28.43

Figure S13: Results obtained from experiment 8; crystallisation solvent: isoamyl alcohol; wash solvent:n- heptane; API grade: special granular; filtration rate (rpm): 100; volume of wash: 2 void volumes; number of washes: 1. a.) Graph showing cumulative API loss in filtrate samples throughout the experiment, mass of PCM API lost during wash = 0 g. b.) Normalised concentration of blue dye impurity in each filtrate sample obtained throughout the experiment. c.) Particle size distribution of the raw paracetamol API and the washed cake sample obtained at the end of the washing experiment, from the damp cake. d.) ^{1}H-NMR analysis results showing the residual crystallisation solvent content in the final washed cake.

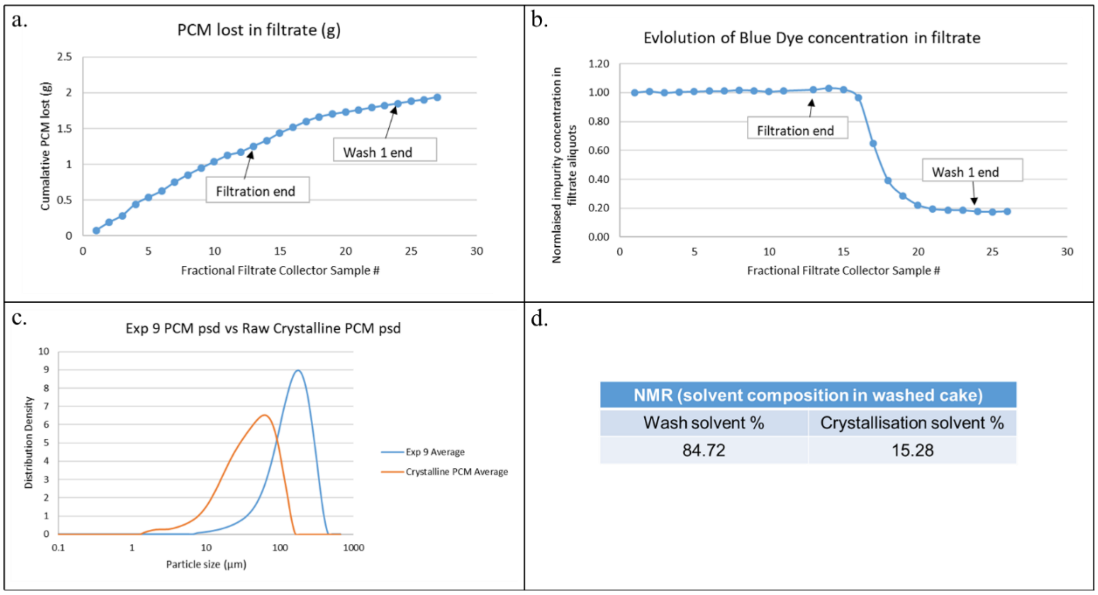

NMR (solvent composition in washed cake)	
Wash solvent %	Crystallisation solvent %
84.72	15.28

Figure S14: Results obtained from experiment 9; crystallisation solvent: isopropanol; wash solvent: acetonitrile; API grade: crystalline; filtration rate (rpm): 10; volume of wash: 2 void volumes; number of washes: 1. a.) Graph showing cumulative API loss in filtrate samples throughout the experiment, mass of PCM API lost during wash = 0.37 g. b.) Normalised concentration of blue dye impurity in each filtrate sample obtained throughout the experiment. c.) Particle size distribution of the raw paracetamol API and the washed cake sample obtained at the end of the washing experiment, from the damp cake. d.) ^{1}H-NMR analysis results showing the residual crystallisation solvent content in the final washed cake.

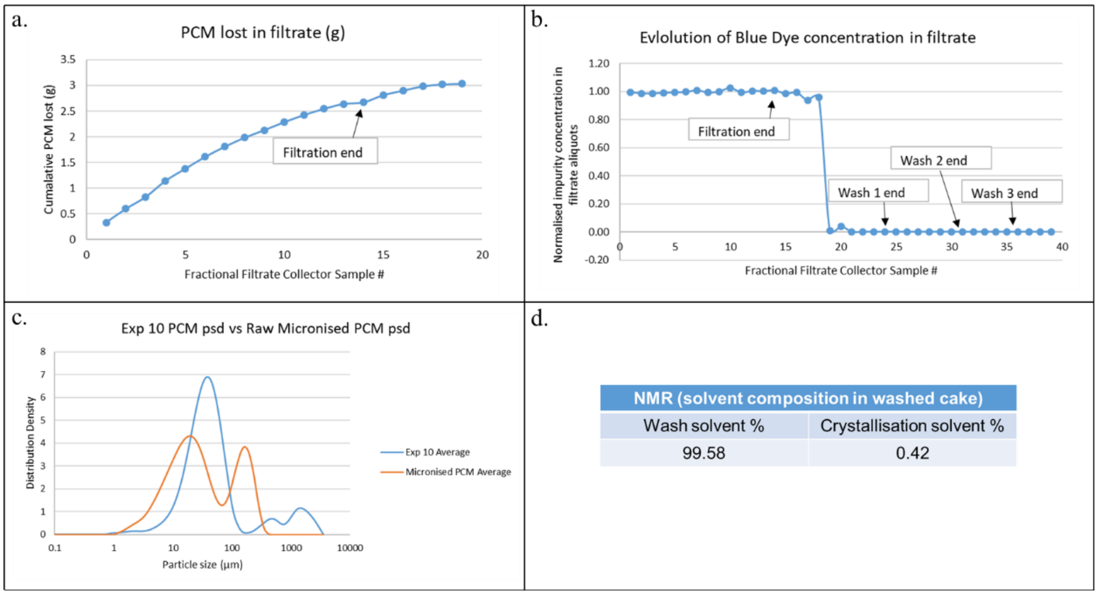

NMR (solvent composition in washed cake)	
Wash solvent %	Crystallisation solvent %
99.58	0.42

Figure S15: Results obtained from experiment 10; crystallisation solvent: ethanol; wash solvent: n-dodecane; API grade: micronised; filtration rate (rpm): 100; volume of wash: 2 void volumes; number of washes: 3. a.) Graph showing cumulative API loss in filtrate samples throughout the experiment, mass of PCM API lost during wash = 0 g. b.) Normalised concentration of blue dye impurity in each filtrate sample obtained throughout the experiment. c.) Particle size distribution of the raw paracetamol API and the washed cake sample obtained at the end of the washing experiment, from the damp cake. d.) ^{1}H-NMR analysis results showing the residual crystallisation solvent content in the final washed cake.

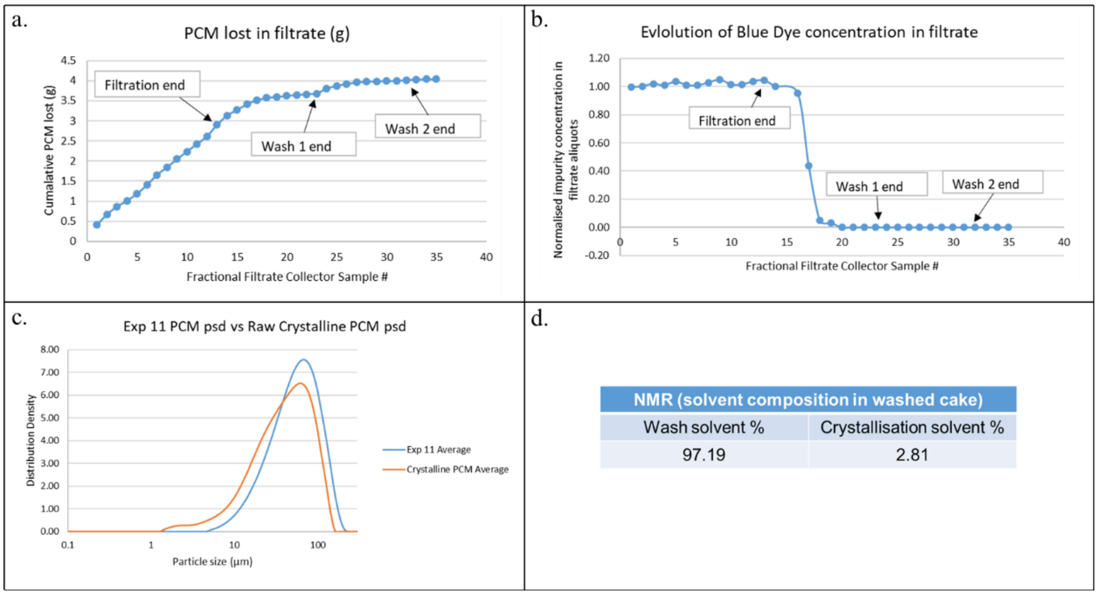

NMR (solvent composition in washed cake)	
Wash solvent %	Crystallisation solvent %
97.19	2.81

Figure S16: Results obtained from experiment 11; crystallisation solvent: ethanol; wash solvent: mix n-heptane solution; API grade: crystalline; filtration rate (rpm): 100; volume of wash: 2 void volumes; number of washes: 2. a.) Graph showing cumulative API loss in filtrate samples throughout the experiment, mass of PCM API lost during wash = 0.31 g. b.) Normalised concentration of blue dye impurity in each filtrate sample obtained throughout the experiment. c.) Particle size distribution of the raw paracetamol API and the washed cake sample obtained at the end of the washing experiment, from the damp cake. d.) ¹H-NMR analysis results showing the residual crystallisation solvent content in the final washed cake.

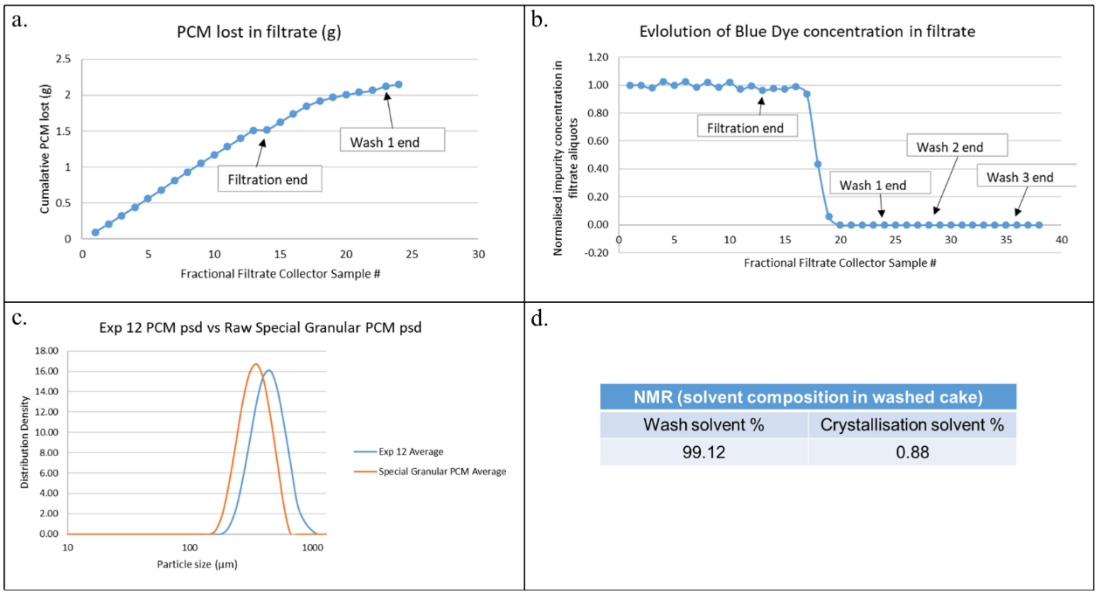

NMR (solvent composition in washed cake)	
Wash solvent %	Crystallisation solvent %
99.12	0.88

Figure S17: Results obtained from experiment 12; crystallisation solvent: isopropanol; wash solvent: mix n-dodecane solution; API grade: special granular; filtration rate (rpm): 10; volume of wash: 2 void volumes; number of washes: 3. a.) Graph showing cumulative API loss in filtrate samples throughout the experiment, mass of PCM API lost during wash = 0.17 g. b.) Normalised concentration of blue dye impurity in each filtrate sample obtained throughout the experiment. c.) Particle size distribution of the raw paracetamol API and the washed cake sample obtained at the end of the washing experiment, from the damp cake. d.) ¹H-NMR analysis results showing the residual crystallisation solvent content in the final washed cake.

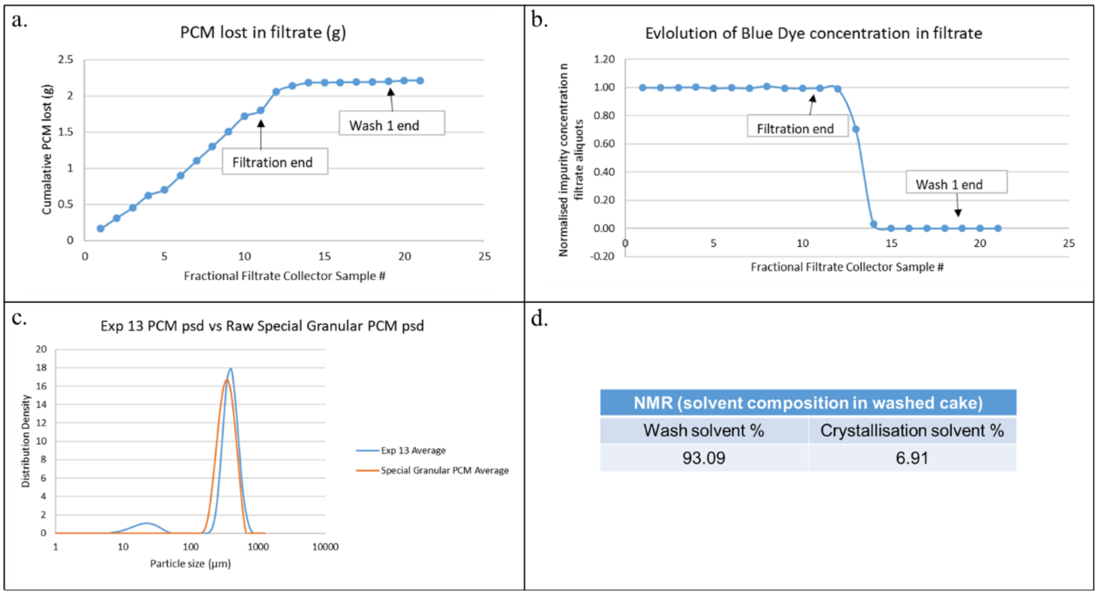

Figure S18: Results obtained from experiment 13; crystallisation solvent: isopropanol; wash solvent: n-heptane; API grade: special granular; filtration rate (rpm): 10; volume of wash: 3 void volumes; number of washes: 1. a.) Graph showing cumulative API loss in filtrate samples throughout the experiment, mass of PCM API lost during wash = 0 g. b.) Normalised concentration of blue dye impurity in each filtrate sample obtained throughout the experiment. c.) Particle size distribution of the raw paracetamol API and the washed cake sample obtained at the end of the washing experiment, from the damp cake. d.) ^{1}H-NMR analysis results showing the residual crystallisation solvent content in the final washed cake.

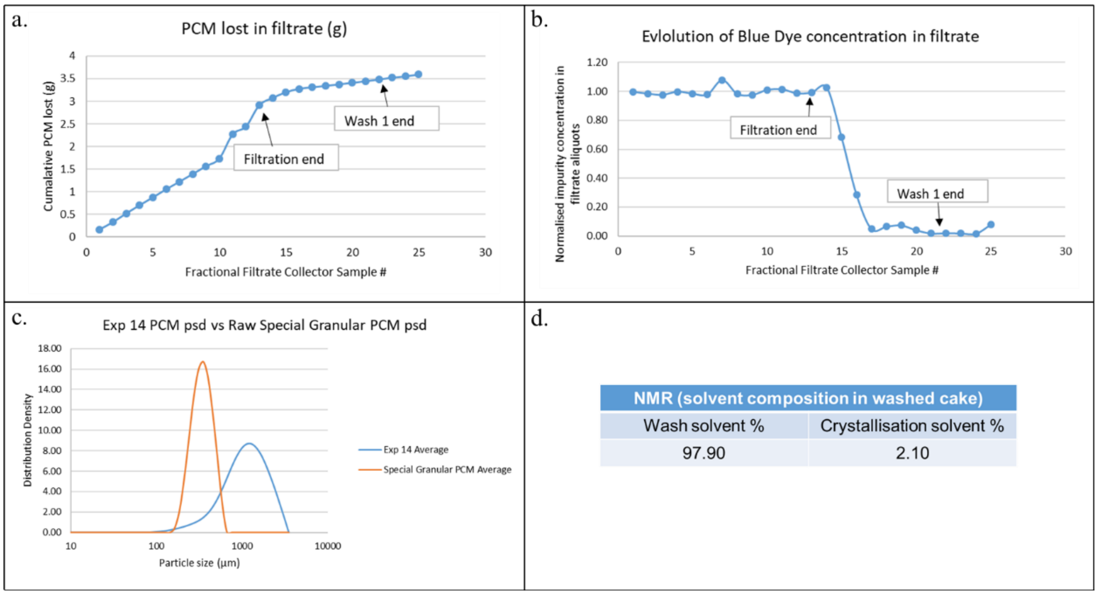

Figure S19: Results obtained from experiment 14; crystallisation solvent: ethanol; wash solvent: acetonitrile; API grade: special granular; filtration rate (rpm): 10; volume of wash: 3 void volumes; number of washes: 1. a.) Graph showing cumulative API loss in filtrate samples throughout the experiment, mass of PCM API lost during wash = 0 g. b.) Normalised concentration of blue dye impurity in each filtrate sample obtained throughout the experiment. c.) Particle size distribution of the raw paracetamol API and the washed cake sample obtained at the end of the washing experiment, from the damp cake. d.) ^{1}H-NMR analysis results showing the residual crystallisation solvent content in the final washed cake.

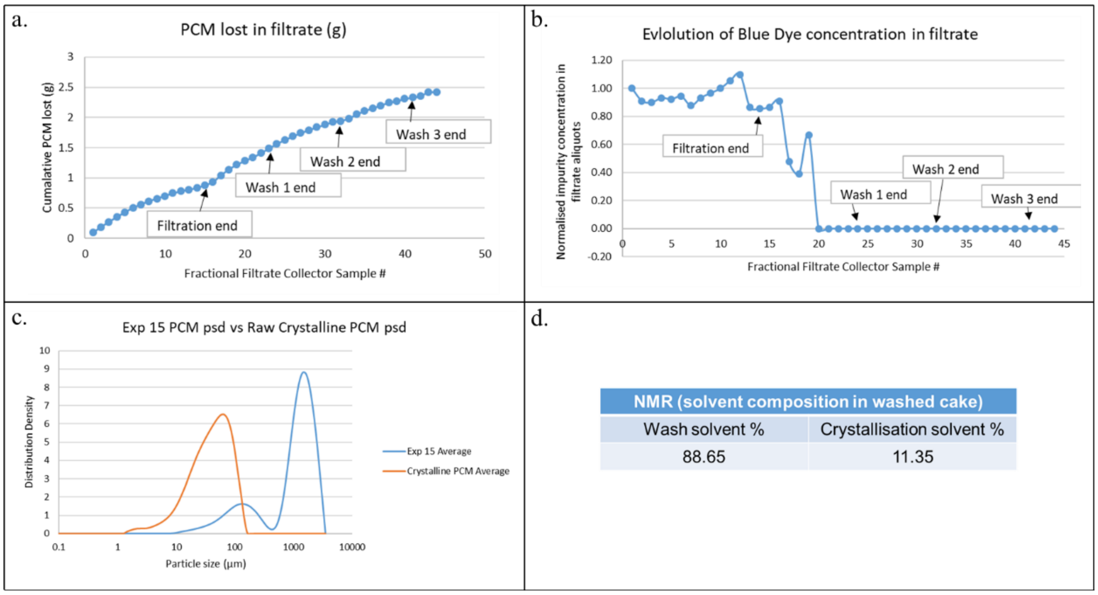

NMR (solvent composition in washed cake)	
Wash solvent %	Crystallisation solvent %
88.65	11.35

Figure S20: Results obtained from experiment 15; crystallisation solvent: isoamyl alcohol; wash solvent: acetonitrile; API grade: crystalline; filtration rate (rpm): 100; volume of wash: 3 void volumes; number of washes: 3. a.) Graph showing cumulative API loss in filtrate samples throughout the experiment, mass of PCM API lost during wash = 1.48 g. b.) Normalised concentration of blue dye impurity in each filtrate sample obtained throughout the experiment. c.) Particle size distribution of the raw paracetamol API and the washed cake sample obtained at the end of the washing experiment, from the damp cake. d.) ^{1}H-NMR analysis results showing the residual crystallisation solvent content in the final washed cake.

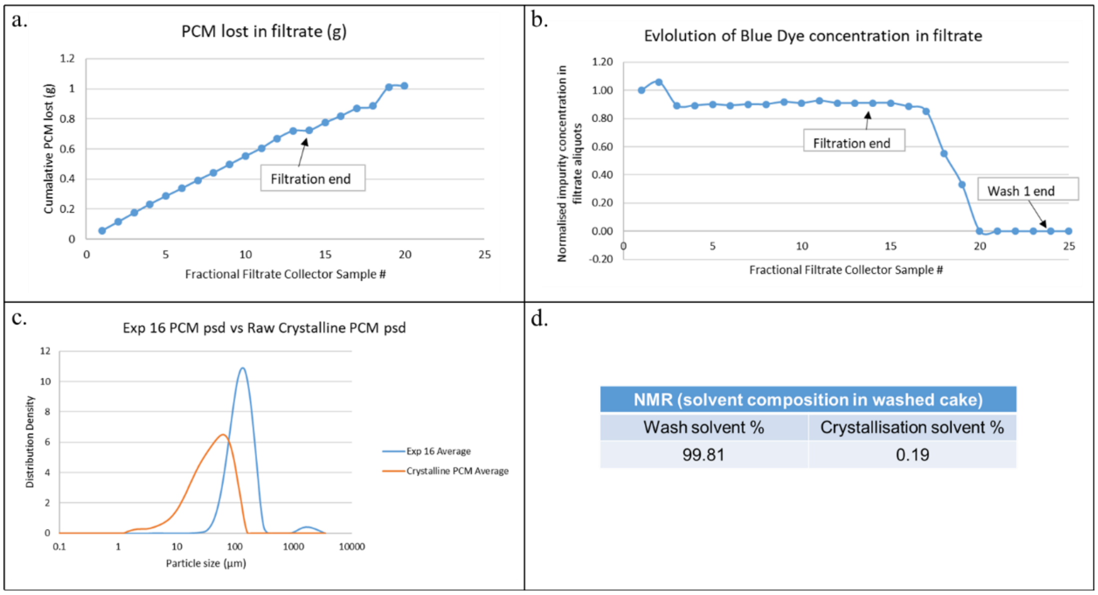

NMR (solvent composition in washed cake)	
Wash solvent %	Crystallisation solvent %
99.81	0.19

Figure S21: Results obtained from experiment 16; crystallisation solvent: isoamyl alcohol; wash solvent: n-dodecane; API grade: crystalline; filtration rate (rpm): 10; volume of wash: 3 void volumes; number of washes: 1. a.) Graph showing cumulative API loss in filtrate samples throughout the experiment, mass of PCM API lost during wash = 0.08 g. b.) Normalised concentration of blue dye impurity in each filtrate sample obtained throughout the experiment. c.) Particle size distribution of the raw paracetamol API and the washed cake sample obtained at the end of the washing experiment, from the damp cake. d.) ^{1}H-NMR analysis results showing the residual crystallisation solvent content in the final washed cake.

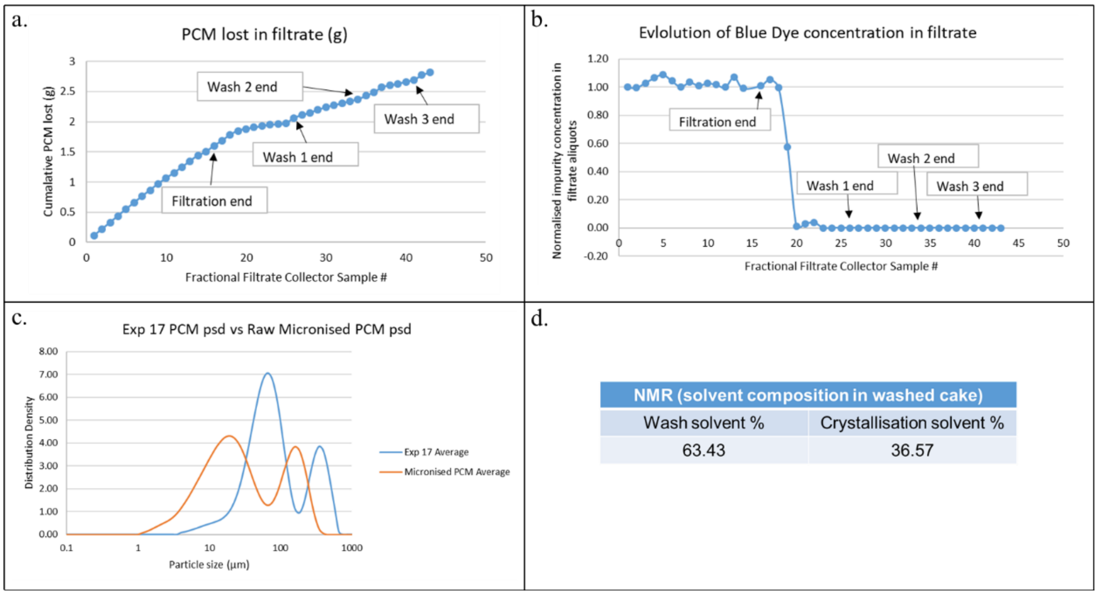

NMR (solvent composition in washed cake)	
Wash solvent %	Crystallisation solvent %
63.43	36.57

Figure S22: Results obtained from experiment 17; crystallisation solvent: isopropanol; wash solvent: mix n-heptane solution; API grade: micronised; filtration rate (rpm): 10; volume of wash: 3 void volumes; number of washes: 3. a.) Graph showing cumulative API loss in filtrate samples throughout the experiment, mass of PCM API lost during wash = 0.95 g. b.) Normalised concentration of blue dye impurity in each filtrate sample obtained throughout the experiment. c.) Particle size distribution of the raw paracetamol API and the washed cake sample obtained at the end of the washing experiment, from the damp cake. d.) ^{1}H-NMR analysis results showing the residual crystallisation solvent content in the final washed cake.

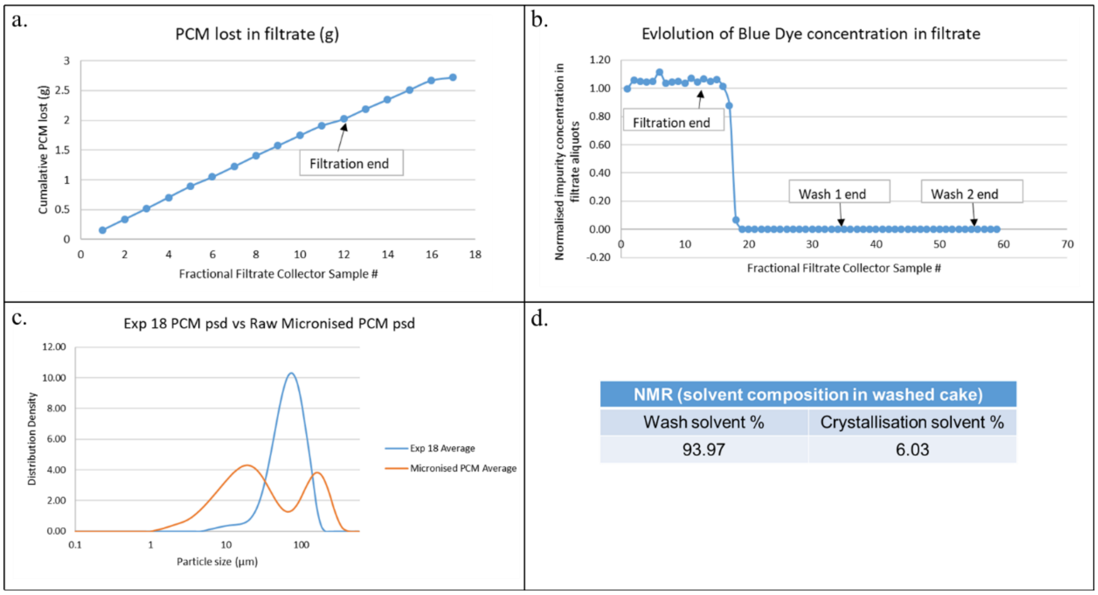

NMR (solvent composition in washed cake)	
Wash solvent %	Crystallisation solvent %
93.97	6.03

Figure S23: Results obtained from experiment 18; crystallisation solvent: ethanol; wash solvent: mix n-dodecane solution; API grade: micronised; filtration rate (rpm): 10; volume of wash: 3 void volumes; number of washes: 2. a.) Graph showing cumulative API loss in filtrate samples throughout the experiment, mass of PCM API lost during wash = 0 g. b.) Normalised concentration of blue dye impurity in each filtrate sample obtained throughout the experiment. c.) Particle size distribution of the raw paracetamol API and the washed cake sample obtained at the end of the washing experiment, from the damp cake. d.) ^{1}H-NMR analysis results showing the residual crystallisation solvent content in the final washed cake.

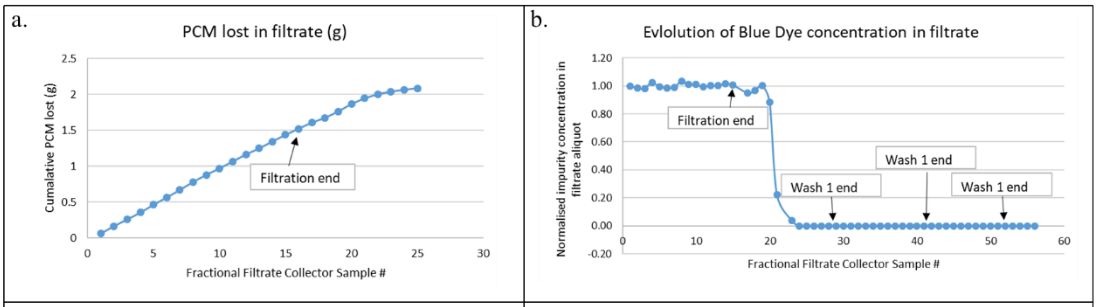

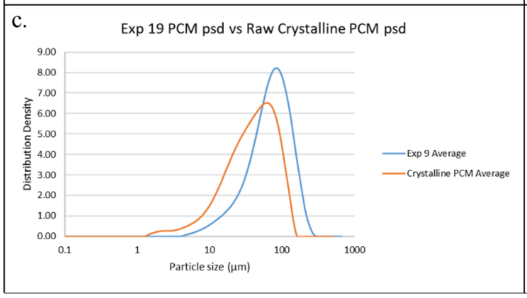

d.

NMR (solvent composition in washed cake)	
Wash solvent %	Crystallisation solvent %
97.93	2.07

Figure S24: Results obtained from experiment 19; crystallisation solvent: isopropanol; wash solvent: mix n-dodecane solution; API grade: crystalline; filtration rate (rpm): 100; volume of wash: 3 void volumes; number of washes: 3. a.) Graph showing cumulative API loss in filtrate samples throughout the experiment, mass of PCM API lost during wash = 0.29 g. b.) Normalised concentration of blue dye impurity in each filtrate sample obtained throughout the experiment. c.) Particle size distribution of the raw paracetamol API and the washed cake sample obtained at the end of the washing experiment, from the damp cake. d.) ¹H-NMR analysis results showing the residual crystallisation solvent content in the final washed cake.

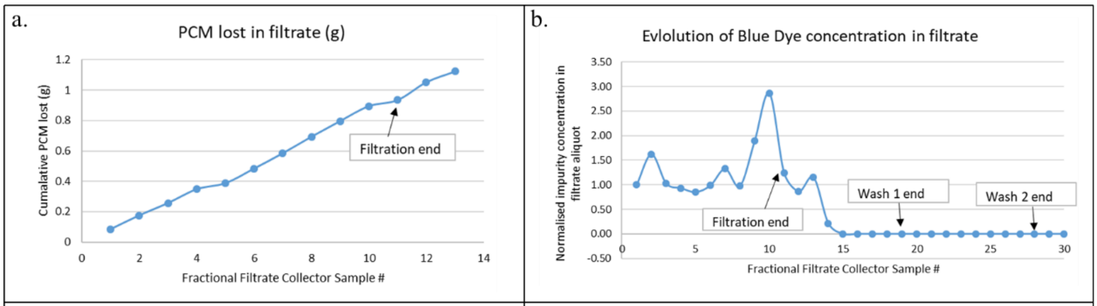

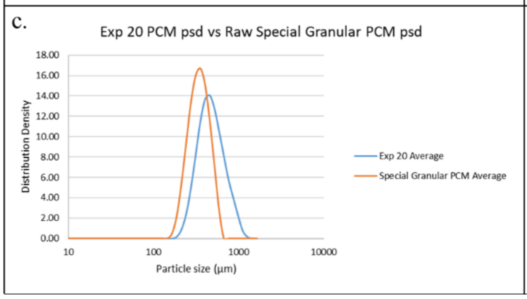

d.

NMR (solvent composition in washed cake)	
Wash solvent %	Crystallisation solvent %
99.51	0.49

Figure S25: Results obtained from experiment 20; crystallisation solvent: isoamyl alcohol; wash solvent: mix n-dodecane solution; API grade: special granular; filtration rate (rpm): 55; volume of wash: 3 void volumes; number of washes: 2. a.) Graph showing cumulative API loss in filtrate samples throughout the experiment, mass of PCM API lost during wash = 0.1 g. b.) Normalised concentration of blue dye impurity in each filtrate sample obtained throughout the experiment. c.) Particle size distribution of the raw paracetamol API and the washed cake sample obtained at the end of the washing experiment, from the damp cake. d.) ¹H-NMR analysis results showing the residual crystallisation solvent content in the final washed cake.

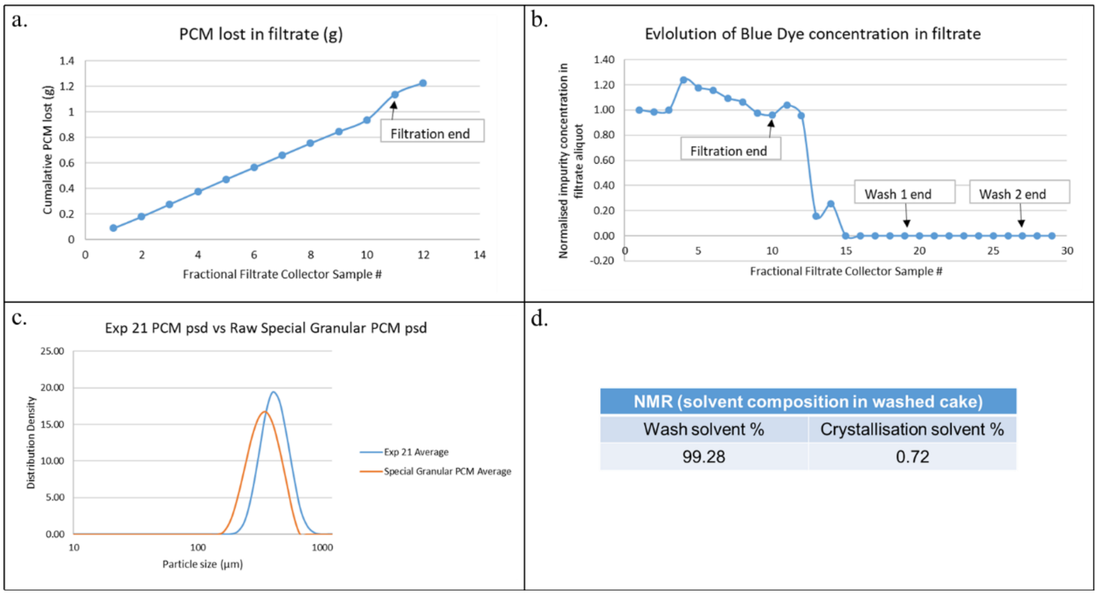

NMR (solvent composition in washed cake)	
Wash solvent %	Crystallisation solvent %
99.28	0.72

Figure S26: Results obtained from experiment 21; crystallisation solvent: isoamyl alcohol; wash solvent: mix n-dodecane solution; API grade: special granular; filtration rate (rpm): 55; volume of wash: 3 void volumes; number of washes: 2. a.) Graph showing cumulative API loss in filtrate samples throughout the experiment, mass of PCM API lost during wash = 0.05 g. b.) Normalised concentration of blue dye impurity in each filtrate sample obtained throughout the experiment. c.) Particle size distribution of the raw paracetamol API and the washed cake sample obtained at the end of the washing experiment, from the damp cake. d.) ^{1}H-NMR analysis results showing the residual crystallisation solvent content in the final washed cake.

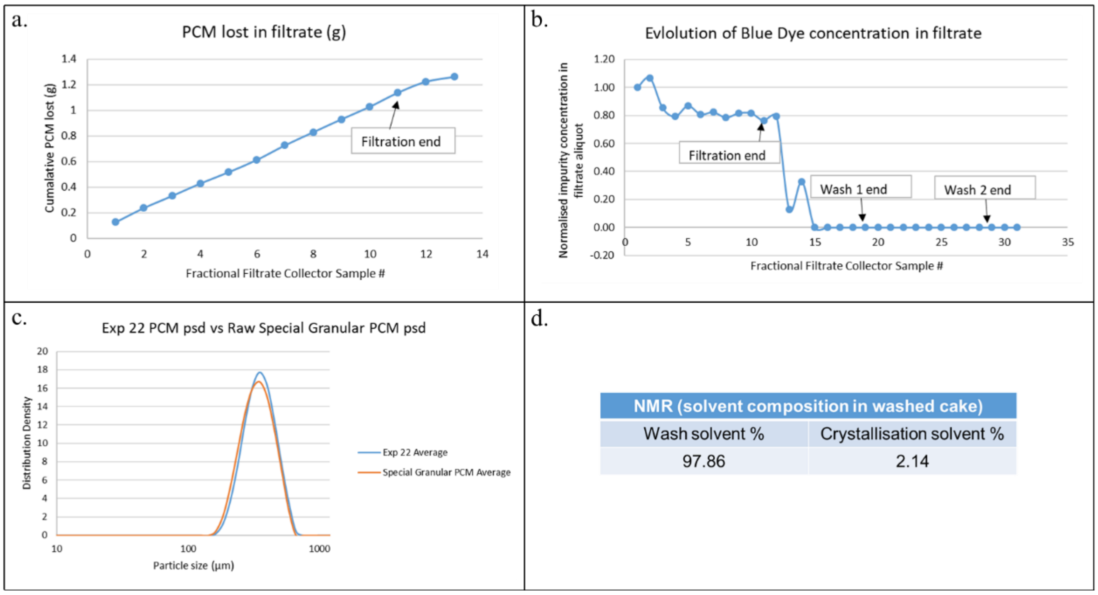

NMR (solvent composition in washed cake)	
Wash solvent %	Crystallisation solvent %
97.86	2.14

Figure S27: Results obtained from experiment 22; crystallisation solvent: isoamyl alcohol; wash solvent: mix n-dodecane solution; API grade: special granular; filtration rate (rpm): 55; volume of wash: 3 void volumes; number of washes: 2. a.) Graph showing cumulative API loss in filtrate samples throughout the experiment, mass of PCM API lost during wash = 0.07 g. b.) Normalised concentration of blue dye impurity in each filtrate sample obtained throughout the experiment. c.) Particle size distribution of the raw paracetamol API and the washed cake sample obtained at the end of the washing experiment, from the damp cake. d.) ^{1}H-NMR analysis results showing the residual crystallisation solvent content in the final washed cake.

References

1. DDBST GmbH. [cited 2021/03/18] http://www.ddbst.com/en/EED/PCP/VIS_C11.php
2. CAMEO Chemicals. [cited 2021/03/01] https://cameochemicals.noaa.gov/chemical/3659
3. Wypych, G. A. Databook of solvents. ChemTec Publishing. **2014**.
4. PubChem. [cited 2020/03/12] https://pubchem.ncbi.nlm.nih.gov/compound/
5. Accudynet. [cited 2020/03/12] https://www.accudynetest.com/visc_table.html
